# Modelling tick abundance and environmental suitability in meso-Mediterranean landscapes for the prevention of tick-borne diseases

**DOI:** 10.1371/journal.pntd.0013741

**Published:** 2025-11-17

**Authors:** Sara Baz-Flores, Alfonso Peralbo-Moreno, Cesar Herraiz, Raúl Cuadrado-Matías, Isabel G. Fernández de Mera, Francisco Ruiz-Fons

**Affiliations:** 1 SaBio Group, IREC (UCLM-CSIC-JCCM), Ciudad Real, Spain; 2 CIBERINFEC, Consorcio de Investigación Biomédica en Red sobre Enfermedades Infecciosas, Instituto de Salud Carlos III, Madrid, Spain; Nanaji Deshmukh Veterinary Science University, INDIA

## Abstract

Ticks are ectoparasites of high sanitary relevance because they host and transmit a multitude of pathogens to vertebrates. A comprehensive understanding of their distribution and abundance is essential for the implementation of effective measures to prevent tick-borne pathogen transmission and tick-borne disease occurrence. Therefore, this study aimed to identify the most environmentally suitable areas and the spatial variation in abundance of exophilic ticks in Castilla-La Mancha (CLM), a highly environmentally diverse meso-Mediterranean region in south-central Spain, where Crimean-Congo haemorrhagic fever is an emerging disease. For modelling tick questing abundance, we performed blanket dragging samplings in 20 sites of CLM from 2019 to 2022. For the environmental suitability modelling, the former survey was complemented with tick presence data from various sources. Along 513 blanket-dragging transects of 100 to 1,200 m long (median: 550 m), we collected 1,260 adult ticks of four species: *Hyalomma lusitanicum, Rhipicephalus bursa, Dermacentor marginatus,* and *Haemaphysalis punctata*. A specific questing abundance index that was estimated for each species and presence data at a 1x1km spatial scale was modelled using generalised linear mixed effects models and MaxEnt, respectively, with selected climatic and habitat variables. We observed that the relevant environmental predictors of tick abundance and suitability showed certain differences and similarities among species. However, in all cases, specific climatic and habitat factors were relevant predictors. The geographical patterns of abundance and suitability also differed among species, with *D. marginatus* and *R. bursa* showing more widespread patterns of both abundance and suitability. In contrast, *H. lusitanicum* displayed higher predicted abundance and environmental suitability in the west of the region, whereas eastern CLM was more suited for the presence and higher abundance of *H. punctata*. This study enhances our understanding of tick ecology in central Spain, offering critical insights for early warning systems and tick-borne disease prevention.

## Author summary

Ticks can transmit serious diseases to humans and animals, making them a public health concern. In this study, we wanted to understand where different types of ticks are most likely to be found and how common they are in Castilla-La Mancha, a region in central Spain. We collected ticks in the field over several years and also gathered data from other sources. Then, we used computer models to predict which areas have the best environmental conditions for ticks to live and where their numbers are likely to be highest. We found that different tick species prefer different parts of the region and different types of environments. Our results help show where the risk of tick bites and tick-borne diseases may be greater. This knowledge can help public health authorities and other stakeholders take preventive actions, such as warning the public or monitoring specific areas more closely.

## Introduction

As obligate hematophagous parasites, ticks are capable of replicating, maintaining and transmitting a vast number of pathogens to their vertebrate hosts. In some regions of the globe, such as the northern hemisphere, ticks can maintain and transmit a greater number of pathogens [1] than the largest global arthropod reservoir of pathogens, the mosquitoes [2]. Many of these pathogens are zoonotic and may cause severe infectious diseases in humans, being therefore of significant concern to the World Health Organisation (WHO). The Crimean-Congo haemorrhagic fever virus (CCHFV), the tick-borne encephalitis virus or the bacteria causing Lyme borreliosis are the most prominent examples of global public health concern [3].

The ecological systems in which zoonotic tick-borne pathogens (TBPs) persist in nature are complex and dependent on a highly variable network of interactions between ticks, pathogens and vertebrates. These interactions are modulated by spatiotemporal variations in local environmental biotic (e.g., habitat composition and structure, vertebrate community structure, host immunity) and abiotic (e.g., soil composition and structure, hydric stress, rainfall regime) conditions [4,5]. Understanding which branches of these complex interaction networks have the greatest influence on the presence and local abundance of the most medically and veterinary relevant tick species can reveal important nodes in these networks on where to act to reduce TBP transmission [6]. Indeed, accurate estimates of the spatial distribution of ticks may provide essential information for preventive action by health authorities in anticipation of disease outbreaks [see 7]. The local abundance of ticks is a crucial parameter in terms of pathogen transmission risk, especially for exophilic (non-nidicolous) ticks that actively seek out hosts [7]. In addition to factors related to human or animal behaviour, tick abundance and the prevalence of TBPs are key drivers of disease incidence in a given area [8]. These factors are directly linked to the risk of tick bites and the likelihood of infection following a bite [9,10]. Since exophilic ticks spend most of their life cycle in natural environments outside their host, local environmental conditions strongly shape their distribution and population abundance patterns [11,12]. Therefore, identifying the environmental conditions most suitable for their presence and abundance can be a useful tool for predicting areas of higher risk of pathogen transmission due to a higher risk of interaction with the ticks that transmit them.

Spain is a country with a high diversity of fauna and flora, where more than 20 species of ixodid ticks have been recorded [13]. Additionally, it is a territory of great environmental contrasts in climate, orography and landscape, which are reflected in a great variety of ecosystems. The influence of the Mediterranean climate in most of peninsular Spain also determines large inter-annual contrasts in environmental conditions that have consequences on the dynamics of ecosystems, consequently influencing the population dynamics of ticks and their hosts [14,15] and the risk of pathogen transmission [8,16]. Castilla-La Mancha (CLM) is an autonomous region of Spain that occupies most of the southern plateau of the country. As a consequence of its extension and variable orography, CLM also presents a great environmental diversity that may determine variations in the diversity and distribution of endemic tick species. It is also a region at risk of CCHFV expansion according to recent findings [17,18] and following the notification of the first human case of CCHF in the region in 2024 [19]; 20 human cases of CCHF have indeed been reported in Spain since 2013 with six deaths [19]. In fact, the most abundant tick species in CLM appears to be *Hyalomma lusitanicum* Koch, 1844 [12,16], one of the primary vectors of CCHFV [20,21]. However, not only is there a risk of CCHF emergence in CLM; other TBPs of veterinary and medical interest that are maintained and transmitted by other tick species are also present in the region [22,23]. Nonetheless, unlike the large number of studies on tick distribution and abundance carried out in northern Spain [5,24–27], studies in the rest of the country generally cover a limited spatial extent [28,29]. As a result, there are still many regions in Spain, including CLM, where the distribution and abundance of tick species remain largely unknown.

Therefore, with the ultimate goal of informing the population as well as health authorities, both medical and veterinary, where in CLM it is more likely to interact and be bitten by one tick species or another, we set two objectives for this study: 1) to reveal the spatial pattern of distribution and abundance of the most sanitary relevant exophilic tick species in the region; and 2) to identify the main environmental conditions that favour these species. Accurately predicting and mapping the spatial distribution and abundance of ticks, while considering the relevant environmental drivers, may provide crucial insights for establishing an early warning system and preventing tick-borne disease (TBD) cases.

## Materials and methods

### Study area

The study was conducted in CLM, a region of 79,463 km^2^ in south-central Spain, which exhibits significant habitat, climatic and orographic contrasts. These contrasts result in marked differences in the local structure of tick communities [30], which may have an impact on the dynamics of different TBPs and on the risk of people suffering from TBDs. The region is characterised by a central zone where agricultural crops predominate, occupying approximately 21% of the territory (16,680 km²), surrounded by less anthropized peripheral areas. In the peripheral areas, both wildlife and extensive livestock are abundant, and coexist in large patches of land [31]. CLM has a continental Mediterranean climate, with cold winters and very hot summers; rainfall is concentrated in the autumn-spring months, and the summers are dry. The region exhibits a certain degree of climatic variation [32], with the southwest exhibiting the highest temperatures and the northeast exhibiting the lowest. The northeast is characterised by a higher cumulative annual rainfall than the rest of the region. The predominant habitat in the western peripheral landscapes of CLM is Mediterranean forest, which features extensive savannah-like areas with abundant oak (*Quercus* spp.) interspersed with scrub and grassland patches. The peripheral mountainous areas of eastern CLM are primarily covered by pine forests with scrub. The northeastern most area, however, is dominated by pure deciduous forests or a mix of deciduous and pine forests.

### Study design and sample collection

The study design contemplated the objectives of knowing both the spatial distribution and the local questing abundance (hereinafter referred to as ‘abundance’) of different non-nidicolous (exophilic) tick species in CLM with a potential impact on animal and human health. Thus, on the one hand, we designed a sampling focused on estimating the local abundance of free-living ticks during host search (questing) and, on the other hand, we completed the information obtained in the first phase with data from different sources on the presence of the most abundant and health-relevant tick species in the region.

#### Tick sampling for questing abundance assessment.

**Fig 1:**
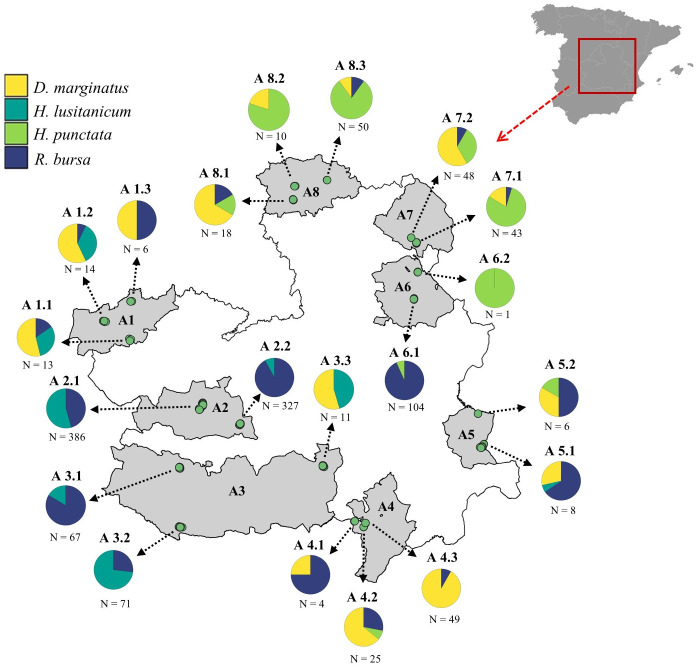
Spatial distribution of sampling sites and the relative proportion of the collected tick species in Castilla-La Mancha. Map showing the spatial distribution of sampling areas and sites for questing ticks across Castilla-La Mancha (lower left panel) and the location of the study region within mainland Spain (upper right panel). Circular charts indicate the proportion of each of the four main tick species per sampling site, relative to the total number of adults collected for those species (other tick species that were collected at low numbers were excluded from these estimates). The total number of adult ticks collected per site is shown below each circular chart. The study areas (A1-A8) are marked in bold type letter case. *Base map source: Junta de Comunidades de Castilla-La Mancha, Datos Abiertos (*https://datos-abiertos-mapasjccm.opendata.arcgis.com/search?tags=OrdenacionTerritorial).

To estimate the local abundance of exophilic ticks, we selected eight areas (A1-A8) in peripheral lands of CLM where ticks might be present and abundant due to the abundance of wildlife [33] and extensive livestock [31] (Fig 1). Two criteria were considered to define the extent of each of these zones, (1) the homogeneity of natural areas in a spatial continuum and (2) the presence of highly humanised environments (agricultural and/or urban) dividing the continuum of natural landscapes. In each of the eight areas, we selected two-to-three sampling points up to a total of 20 throughout CLM (Fig 1 and S1 Table). For the selection of these points, we divided CLM into a grid of 10 x 10 km UTM plots. In each plot we estimated the density of hunted wild ungulates per km^2^ based on hunting bag data collected at the hunting ground scale by the environmental authorities of the regional government [see 33]. Additionally, we used data from the livestock census conducted in Spain in 2009 and available at the Spanish Statistics Institute [www.ine.es; see [34]] at the municipal scale to estimate the density of domestic ruminants (cattle, sheep and goats) in each of the 10 x 10 km UTM plots of CLM [see methodological details in 17]. Finally, from the set of 10 x 10 km plots included in each of the eight sampling zones, we selected the two-to-three plots where the densities of hunted wild ungulates and domestic ruminants were highest. For the final selection of sampling points within each of the 10 x 10 km plots, we visualized the orography and structure of the landscape on recent (2018) Google Earth orthophotos. We identified two-to-four potential sampling points per 10 x 10 km plot that were accessible and potentially attractive to wildlife, e.g., the presence of water in the form of streams, rivers or water bodies was considered an attractive feature for wildlife due to the significant water scarcity during the period of questing activity of exophilic ticks in southern Spain (May-October) [12,16]. For the selection of the definitive sampling points among the potential ones selected, they all were visited to corroborate the presence of wildlife species based on direct observations (birds) and on indirect signs of presence (droppings, scat, tracks and other signs of presence) and the adequacy of the area for dragging the vegetation with a cotton blanket to estimate tick abundance [see 9].

We focused our sampling on questing ticks rather than on ticks collected from hosts. Estimates of host-attached tick abundance require intensive sampling efforts, standardized host capture protocols, and sufficient sample size per host species to yield robust and comparable results. While this approach has been successfully applied by our group at small spatial scales [see 7], extending it to large spatial and temporal scales would have required a substantially higher logistic and financial effort. Focusing on questing ticks allows standardized comparisons between sites and has proven to be a reliable approach for estimating spatial variation in tick abundance when consistent sampling is applied [5].

This study was conducted within the scope of two different research projects, resulting in variations in the temporality of sampling areas A2 and A3 compared to the rest of the areas. In these two areas, biweekly samplings were conducted from March to December between 2019 and 2021. In the rest of the areas, sampling was carried out on a monthly basis during the two periods of higher questing activity of the adults of the exophilic tick species present in CLM [30], March-June and October-November [12,16], between 2020 and 2022. A total of 513 samplings were carried out in all eight areas between 2019 and 2022. Each sampling consisted of random transects of variable length (from 100 to 1,200 meters) within which 10 m-long blanket drags were carried out with a 1 m^2^ white cotton flannelette [35]. The sampling unit considered to perform the models was the transect. Each transect track was recorded with a hand-held GPS device. All the ticks captured in each 10 m blanket drag were collected into 1.5 mL tubes and transported to our laboratory, where the ticks were identified to the species level based on morphological characteristics under a stereoscopic microscope (Leica S9E) following [13] and preserved frozen at -80ºC.

#### Data collection for environmental suitability assessment.

We assembled occurrence records within CLM for four tick species: *Hyalomma lusitanicum, Rhipicephalus bursa, Dermacentor marginatus* and *Haemaphysalis punctata*. We selected these species as they were the most frequently encountered during the survey of questing ticks and in the available scientific literature [30,36,37]. For the suitability models, we considered records from all life stages (larvae, nymphs, and adults) and considered both records of ticks in vegetation and ticks on hosts. Tick occurrences were obtained from the data obtained in questing tick abundance estimates in the eight study areas, from previously peer-reviewed literature [16,29], the Global Biodiversity Information Facility (GBIF; www.gbif.org) and information retrieved in the course of epidemiological and ecological studies conducted by our group since 2000. We collected an initial total of 1,123 occurrence points for the four tick species from the diversity of available sources. The records were subjected to a rigorous examination, with only those bearing clear geographic references being considered. Duplicate records were eliminated. Tick presences were denoted within a 1 km^2^ grid. Thus, if multiple presence points coincided within a 1 km^2^ grid cell for the same tick species, this was considered as a single presence point for the model, thereby omitting any redundant occurrence records to remove spatial bias in estimating ecological niche models [38]. We considered the centroid of the 1 x 1 km grid cell as the presence point. Finally, this study employed 316 geo-referenced records of presence of the four tick species.

### Environmental predictors

To predict the most environmentally suitable areas for the presence and abundance of the four tick species considered in this study, we performed statistical modelling with environmental predictors (S2 and S3 Tables). For abundance tick modelling with local environmental traits, we created a 1 km radius circular buffer (313.7 hectares) around the central point coordinate of each transect. The buffer size was selected by considering the maximum length of the transects, accounting for potential inaccuracies resulting from the GPS margins of error, and to cover the extent of the ticks’ hosts (lagomorphs, carnivores and ungulates) [29,30,36,39] range in Mediterranean environments [see 7]. The estimated predictor values were rescaled to the spatial scale of the selected buffer by weighted averaging. For environmental suitability modelling, the predictors were rescaled to a UTM 1 x 1 km spatial resolution scale.

Data from the WorldClim 2 project database (https://worldclim.org/version2) were used to characterise the climate locally with the aim of modelling environmental suitability for tick presence/abundance at the spatial scale of CLM, for which climatic rather than weather data might be more appropriate to characterise long-term local abiotic traits [see 18]. The data were downloaded with a spatial resolution of 1 km^2^. The WoldClim data includes 19 bioclimatic variables originally derived from monthly temperature and precipitation, solar radiation, windspeed and vapour pressure [40]. Bioclimatic variable 3 (Isothermality) was omitted from the analysis, as it is not relevant to the type of area being studied. We also included the Normalized Difference Vegetation Index (NDVI) due to its significance in shaping the ecological niche of ticks [41] and its local abundance [11]. The NDVI serves as an indicator of plant photosynthetic activity, which is related to soil water availability and, consequently, reflects the hydric stress experienced by off-host ticks [42,43]. NDVI data were downloaded from the MODIS website (https://modis.gsfc.nasa.gov) for the period 2019–2022 at a 250 m spatial and 16 days temporal resolutions. We calculated the annual, summer, winter, spring and autumn mean and the annual variance of the NDVI, across all layers covering the period January 2019-December 2022.

The local structure of the habitat can affect tick survival and activity patterns by modulating adverse weather conditions, e.g., hydric stress [44]. It also influences ticks’ host space usage due to variations in available resources and shelter [45]. Land use data were obtained from the SIOSE 2017 database with a resolution of 250 m (www.siose.es). We considered six land cover predictors for the abundance analysis i) grassland; ii) shrubland; iii) deciduous broadleaf forest; iv) evergreen broadleaf forests; v) coniferous forest; and vi) woodland. The suitability analysis also encompassed categories pertaining to crops and other land uses that are unfavourable for ticks; that is, eight categories in total. The land use predictors were employed as continuous predictors in tick abundance modelling, whereas in environmental suitability analyses, a categorical variable was created with each of the previously described land uses as individual categories. Therefore, in the abundance analyses we worked with the percentage of each land use present in each buffer, while in the case of suitability analyses, each 1 x 1 km UTM grid cell takes the value of the land use type that represents the highest proportion within that cell.

### Tick abundance modelling

We standardized and rescaled all continuous predictors using the ‘scale’ function in the R statistical software before any statistical analysis. This process helped reducing the variability in measurement scales across the diversity of estimated potential predictors. The variables having Pearson correlation coefficient values (r), r > |0.70| (significant at alpha = 0.05) within the set of estimate predictors were grouped together according to hierarchical cluster analysis (S1 Fig). One predictor from each cluster was selected based on the calculated Variable Inflation Factors (VIF), to avoid the multi-collinearity among the data and associate statistical trouble for output interpretation [46]. VIF values were calculates using the ‘vif’ function of the R package ‘usdm’ [47]. The variables with VIF < 10 were included in the statistical modelling of tick presence and abundance. All variables considered for abundance and environmental suitability modelling are described in S2 and S3 Tables.

Abundance models for *H. lusitanicum, R. bursa, D. marginatus* and *H. punctata* were built using the glmmTMB R package [48]. We focused on these four tick species because they were the most abundant questing adult species collected in vegetation during field surveys and are of particular epidemiological and ecological relevance in the study region. These models were built using only adult specimens. There were several reasons for using adults, including (1) that adults of the four species are the most frequent stage biting humans, (2) that the sampling design is more representative for estimating adult that immature abundance due to the lower spatial aggregation of the former [44], (3) that some of the immatures of these species are endophilic or both larvae and nymphs feed on the same individual (two-host cycle), and (4) because our temporal sampling design may not have accurately estimated immature abundance, both because of their short activity period and because the immatures of some species are active outside the sampling period. Generalized linear mixed effects models (GLMMs) with a negative binomial distribution (‘nbinom2’ family; log link function) were performed due to high levels of overdispersion in the response variable [49]; zero-inflation was discarded using the ‘testZeroInflation’ function of the R package ‘DHARMa. The response variable for abundance modelling was the number of adult ticks (*H. lusitanicum, R. bursa, D. marginatus* or *H. punctata*) captured per transect. In order to account for the variation in sampling efforts between transects, we included the logarithm of the length (in meters) of the transects as an offset. The sampling season of the year was considered as a random effect to build abundance models. The final models were obtained through a forward stepwise procedure based on the Akaike Information Criteria (AIC) [50] using the ‘buildglmmTMB’ function of the R package ‘buildmer’ [51]; the lower the AIC value, the better the fit of the model. The structure of final model residuals was explored using the ‘simulateResiduals’ and ‘testUniformity’ functions of the R package ‘DHARMa’ [52]. The spatial autocorrelation of the residuals of the best-fit model was evaluated using the ‘testSpatialAutocorrelation’ function of the R package ‘DHARMa’ [52]. The predictive capacity of the models was evaluated using the train-test split methodology. The data set was partitioned into a 70% training set, which was employed to parameterize the model, and a 30% test set, which was used to assess the predictive performance of the model on independent data [53]. We performed correlation tests and calibration plots to estimate the association between observed and predicted abundance values on the test data set. Calibration plots were generated by dividing the predicted tick abundance for the test dataset into nine percentile bins. The mean predicted abundance for each bin was then plotted against the mean observed abundance for the corresponding localities. This procedure was repeated one hundred times, allowing us to calculate the mean and confidence interval of correlation values, thus offering a comprehensive evaluation of the model’s predictive accuracy [11]. Finally, the models were employed to forecast spatial patterns of tick abundance across the entire study area, with a spatial resolution of 1 x 1 km. The reference season used for model projection depended on the tick species studied; we employed the season with the highest number of ticks of the species of interest as the reference for model spatial projection. Thus, spring was used for *H. lusitanicum*, autumn for *D. marginatus* and *H. punctata*, and summer for *R. bursa* (S2 Fig). The model projection was estimated using the ‘predict’ function of the R package ‘car’ [54].

### Suitability models

For each tick species, environmental suitability models were constructed using the environmental predictors and local presence data of the species in CLM. The MaxEnt model v3.3.3 [55] was used through the ‘dismo’ R package for modelling [56]. The occurrence data were randomly divided in the ratio 80:20 for training and testing of the model, respectively. The MaxEnt models were run with 10-fold cross validation to mitigate the uncertainty and biases associated with the predictions. The background points were set to 1,000 within the extent of the study area. To prevent overfitting of the model, a range of regularisation coefficient values (0.1-3.0, with a difference of 0.1) was explored and the value that yielded the highest model performance was selected, based on the area under the curve - AUC [57]. The selected values were 0.5 for the *H. lusitanicum* model, 0.4 for the *R. bursa* model, 0.4 for the *D. marginatus* model and 1.6 for the *H. punctata* model. The rest of the model parameters were kept as default. The final results were derived from the average of multiple cross-validation runs, yielding values between 0 and 1. These values can be interpreted as the probabilities of occurrence. The suitability curves for each predictor were computed, and the contribution of each predictor to the habitat suitability models was determined using the Jackknife test bar chart and the predictor contribution table. The outcome of the Jackknife test indicates the extent of the gain derived from the combined influence of all variables, as well as the isolated impact of each variable. The presence of a greater gain for an individual variable indicates that the variable contributes more information to the understanding of species distribution [58]. The suitability models were evaluated based on computed AUC and average omission and prediction statistics. AUC value of 1 indicates the best run, while a value of 0.5 indicates that the model is close to random and the accuracy is low [55].

### Multivariate Environmental Similarity Surfaces (MESS)

**Fig 2:**
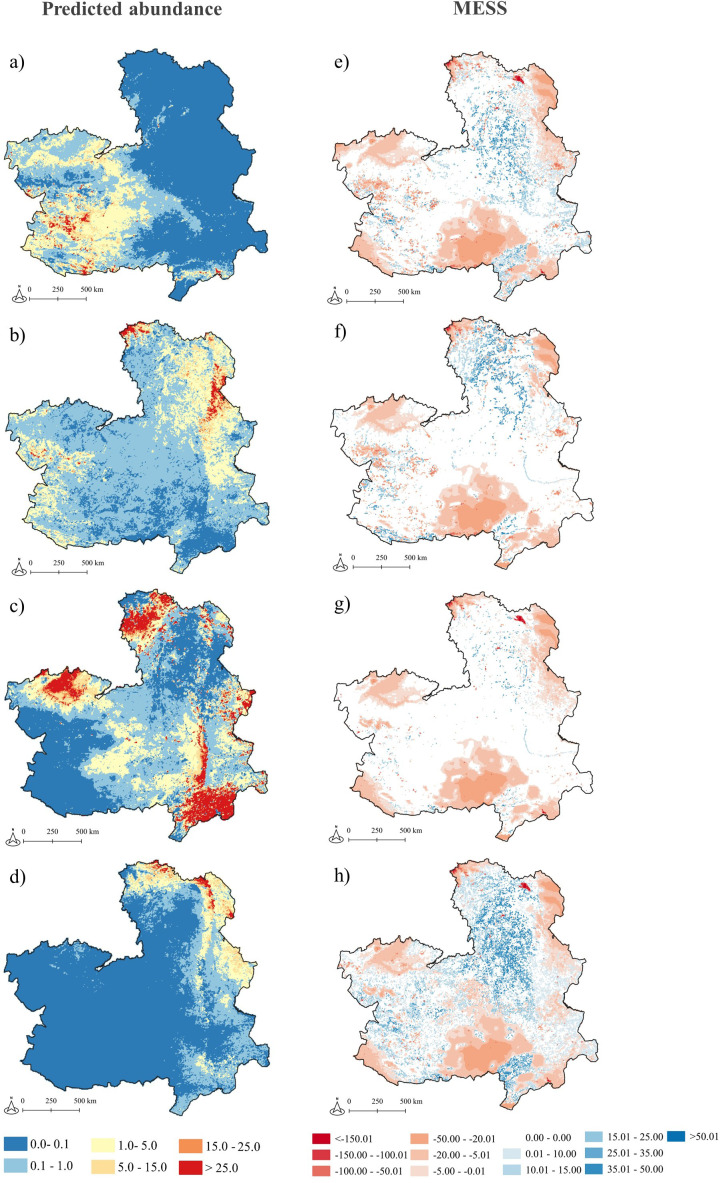
Projection of tick abundance models of four tick species in Castilla-La Mancha. Spatial projection at 1 km^2^ spatial resolution of adult tick questing abundance models (left) and of the Multivariate Environmental Suitability Surfaces (MESS) of the models (right) to Castilla-La Mancha region. From top to bottom: *Hyalomma lusitanicum* (a,e), *Rhipicephalus bursa* (b,f), *Dermacentor marginatus* (c,g) and *Haemaphysalis punctata* (d, h). Warmer colours in the abundance maps indicate higher tick abundance, expressed as the expected number of adult ticks per sampling transect. In the MESS maps, warmer colours (pink to red) represent areas where predictive modelresults are not extrapolable, whereas white and blue colours show the areas where environmental conditions are within the range of the training dataset. *Base map source: Junta de Comunidades de Castilla-La Mancha, Datos Abiertos (*https://datos-abiertos-mapasjccm.opendata.arcgis.com/search?tags=OrdenacionTerritorial).

The selected models were employed to predict tick abundance in locations that had not been sampled, requiring the development of a measure of similarity between new environments and sampled areas. A key indicator for evaluating and interpreting the quality of prediction maps is the spatial representation of the model’s level of extrapolation relative to the sampling pattern. The MESS index indicates the similarity of a location to the set of reference locations, with respect to the chosen predictors [59]. The index allows negative values, which indicate areas where at least one variable falls outside the distribution of the reference points. Such areas are designated as novel environments. Thus, when assessing similarity, we focus on the positive values of this index. The higher the value, the more similar the new environment to the sampled ones [59]. We calculated MESS for abundance models using the ‘MESS’ function of the ‘modEVA’ R package [60] and the results were represented in a 1x1 km grid (Fig 2). For suitability models, we did not calculate MESS because training and projection were restricted to the same region. The 1000 background points used in model calibration adequately covered the environmental space of the study area, ensuring representativeness and making the calculation of MESS unnecessary.

## Results

### Questing ticks captured and estimated abundance

A total of 513 transects were conducted, resulting in the collection of 11,049 ticks, encompassing all life stages (larvae, nymphs, and adults). For the purposes of this study, however, only adult ticks were included in the analyses. Of these, 1,260 were adults of the tick species under study. In addition, a small number of other tick species were also collected, including *Rhipicephalus sanguineus* sensu lato and *Ixodes ricinus*, both in the vegetation and on hosts, but their low abundance prevented them from being taken into account for modelling. Of these 1,260 adult ticks, 313 were classified as *H. lusitanicum*, 691 as *R. bursa*, 144 as *D. marginatus*, and 112 as *H. punctata* (S1 Table). The number of tick captures was very heterogeneous across areas (range 12.74 - 153.28 ticks/ha; S4 Table), as well as within sampling points within the same area (S4 Table). The distribution of the different tick species also varied between areas. For instance, *H. lusitanicum* was mainly captured in the western part of CLM, while *H. punctata* was mainly found in the northeastern part of the region. In contrast, *D. marginatus* and *R. bursa* were captured in most of the sampled areas (Fig 1). Differences were also observed in the number and species of ticks captured in different seasons of the year (S5 Table and S2 Fig). Thus, the highest number of *H. lusitanicum* was captured in the spring (March-May), *R. bursa* in the summer (June-August), and *D. marginatus* and *H. punctata* in the autumn (September-November) (S5 Table and S2 Fig).

### Tick presence data for environmental suitability models

**Fig 3:**
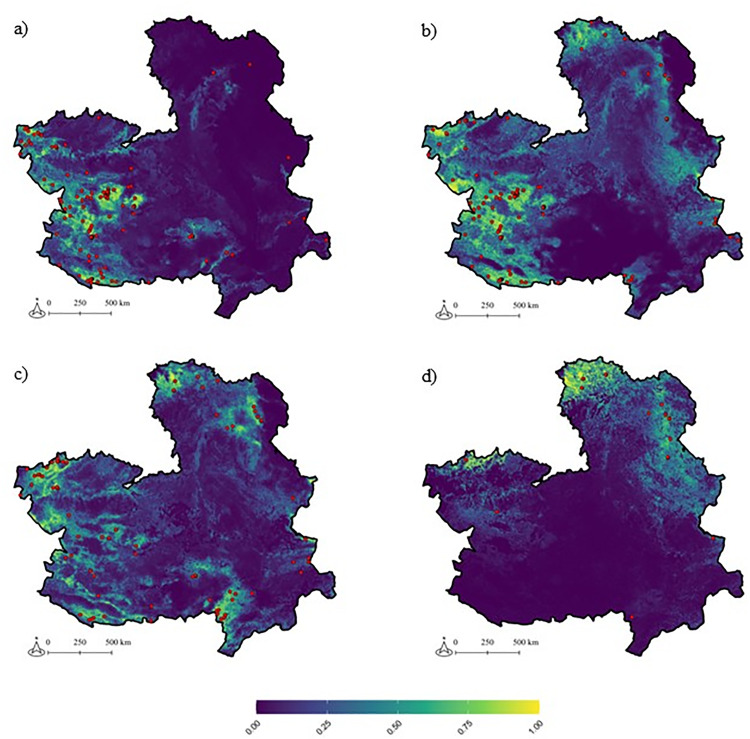
Environmental suitability and model extrapolation for adult ticks in Castilla-La Mancha. Predicted suitable environment from MaxEnt models for the adults of the four tick species studied: *Hyalomma lusitanicum* (a), *Rhipicephalus bursa* (b), *Dermacentor marginatus* (c) and *Haemaphysalis punctata* (d). Spatial resolution of the maps is 1 km^2^. The red dots are the centroids of the 1 km^2^ square of species presence after cleaning and thinning. Blue colours indicate areas of low suitability, while yellow colours represent areas of high suitability. *Base map source: Junta de Comunidades de Castilla-La Mancha, Datos Abiertos (*https://datos-abiertos-mapasjccm.opendata.arcgis.com/search?tags=OrdenacionTerritorial).

We assembled 316 geo-referenced records of the four tick species studied (Fig 3). Of these, 113 records corresponded to the presence of *H. lusitanicum*, 103 to *R. bursa*, 84 to *D. marginatus*, and 16 to *H. punctata.* Similarly to the results of questing tick sampling, we observed a higher presence of *H. punctata* in the northeast of CLM, and *D. marginatus* appeared to be more evenly distributed across the peripheral areas of the region. For *H. lusitanicum* and *R. bursa*, we observed presences across various peripheral areas of the region but mainly in western parts of the region (Fig 3).

### Tick abundance models

Variables selected to perform final models are shown in S2 Table. The output of the best-fit statistical tick abundance models is summarised in Table 1. The selected predictors and their positive/negative relationship with tick abundance varied between the models. Thus, annual precipitation and precipitation seasonality were the most influential predictors of *H. lusitanicum* abundance. The model indicated a negative association with annual precipitation and a positive one with precipitation seasonality,. In the case of the *R. bursa* model, the predictor with the strongest relationship with this tick species abundance was annual precipitation, However, in contrast to the *H. lusitanicum* model, it demonstrated a positive relationship with the abundance of *R. bursa.* Annual precipitation was also the strongest predictor of *D. marginatus* abundance and as in the case of the *H. lusitanicum* abundance model, showed a negative relationship with its abundance. For *D. marginatus*, deciduous broadleaf forest, a habitat-related predictor, was identified as a significant factor influencing the abundance of the species, exhibiting a positive relationship. Finally, the abundance model for *H. punctata* indicated that the most relevant factor for the abundance of this species was the seasonality of precipitation, which, in contrast to *H. lusitanicum*, showed a negative relationship with abundance (Table 1). These associations should be interpreted with caution, as precipitation variables may reflect the influence of other correlated environmental factors (e.g., temperature, vegetation productivity), and the relationships captured by the models may not represent direct causal effects.

**Table 1:** Output of the best fitted models (Generalized Linear Mixed Effects Models) built for questing adult tick abundance of the four studied tick species.

Model	Predictor	Estimate	SE	z	p^a^
*H. lusitanicum*	*Intercept*	-10.9709	1.1354	-9.663	***
	Annual median diurnal range	-1.8482	0.3065	-6.030	***
	Annual precipitation	-3.0396	0.3990	-7.618	***
	Precipitation seasonality	3.9330	0.5777	6.808	***
	Coniferous forest cover	0.5539	0.2633	2.103	*
	Temperature seasonality	1.0760	0.3018	3.565	***
	Shrubland cover	-0.4794	0.1869	-2.565	*
	Woodland cover	0.3229	0.1502	2.149	*
*R. bursa*	*Intercept*	-8.4655	1.1274	-7.509	***
	Annual median diurnal range	-0.9056	0.1783	-5.080	***
	Annual precipitation	1.1592	0.1759	6.590	***
	Deciduous broadleaf forest cover	-0.6963	0.1630	-4.149	***
	Shrubland cover	-0.5651	0.1528	-3.697	***
	Mean temperature of wettest quarter	0.6096	0.1512	4.031	***
*D. marginatus*	*Intercept*	-11.6007	1.1619	-9.984	***
	Precipitation seasonality	-2.6188	0.3639	-7.197	***
	Coniferous forest cover	1.4933	0.2221	6.723	***
	Annual median diurnal range	1.9554	0.3606	5.422	***
	Annual precipitation	-3.6783	0.6277	-5.860	***
	Mean temperature of wettest quarter	-1.5097	0.3133	-5.078	***
	Deciduous broadleaf forest cover	3.0261	0.6935	4.363	***
	Woodland cover	-0.7571	0.2041	-3.708	***
*H. punctata*	*Intercept*	-12.3956	0.9617	-12.889	***
	Precipitation seasonality	-3.4973	0.5317	-6.577	***
	Mean temperature of wettest quarter	-1.0515	0.2266	-4.641	***
	Annual NDVI variance	-1.1679	0.6971	-1.675	0.09
	Woodland cover	-0.3505	0.1883	-1.861	0.06
	Annual median diurnal range	0.7449	0.3164	2.354	*
	Annual precipitation	-0.6071	0.2970	-2.044	*

^a^p-value (*p < 0.05, **p < 0.01, ***p < 0.001).

The spatial projection of the models showed differences in the distribution of the areas of highest abundance of the different tick species. Therefore, the best-fit models predicted the highest abundance of *H. lusitanicum* adult ticks in western CLM (within areas of low uncertainty in model prediction), whereas the highest abundance of *H. punctata* was predicted in the northeast of the region. Conversely, the *R. bursa* and *D. marginatus* highest abundance areas showed a more uniform distribution across CLM, particularly *R. bursa*. In terms of uncertainty, the four models displayed a comparable pattern, with the areas of greatest uncertainty situated in the peripheral regions of CLM, extending through the central and southern parts of the region. In contrast, the areas of lowest uncertainty, where the model extrapolation is more reliable, exhibited some discrepancies between the models. In the *H. lusitanicum* model, large areas of low uncertainty were identified in the eastern portion of the region, with some also appearing in the western section. Regarding *R. bursa*, low uncertainty areas were identified in the western part of CLM, which corresponded to those identified in the *H. lusitanicum* model. Nevertheless, the majority of areas exhibiting low uncertainty were located in the northeastern part of the region. The *D. marginatus* model exhibited the fewest areas of low uncertainty, which were primarily located in the northeast and southern parts of CLM. The *H. punctata* model exhibited the greatest extent of low uncertainty, encompassing the majority of CLM with the exception of select central and peripheral regions (Fig 2).

We observed normal distribution and dispersion patterns of the residuals for the four models (S3 Fig). No spatial correlation was found, as indicated by the non-significant DHARMa Moran test results in all cases (p = 0.45 for *H. lusitanicum*; p = 0.11 for *R. bursa*; p = 0.99 for *D. marginatus* and p = 0.06 for *H. punctata*). Pearson correlation coefficients of the observed and predicted abundance correlation tests were 0.61 (95% confidence interval [95%_CI_]: 0.56-0.64) for *H. lusitanicum*, 0.55 for *R. bursa* (95% CI: 0.49-0.59), 0.60 for *D. marginatus* (95% CI: 0.22-0.86) and 0.63 for *H. punctata* (95% CI: 0.26-0.86) (S4 Fig).

### Habitat suitability models

Predictors selected to perform the MaxEnt models are shown in S3 Table. The Area Under the Curve (AUC) value for the habitat suitability models was found to be between 0.790 ± 0.058 for *H. lusitanicum* and 0.750 ± 0.048 for *R. bursa* (S5 Fig), indicating a moderate level of success in predicting the habitat suitability distribution of the species. The omission and predicted area plot (S5 Fig) with the choice of cumulative threshold demonstrates that the omission rate on test data aligns with the predicted omission rate for test data drawn randomly from the MaxEnt distribution, with a high degree of accuracy, in the four models. This indicates that the models are well-calibrated and generalise adequately to the test data. The width of the band representing the mean omission ± one standard deviation suggests high robustness in model predictions, except for the *H. punctata* model.

**Fig 4:**
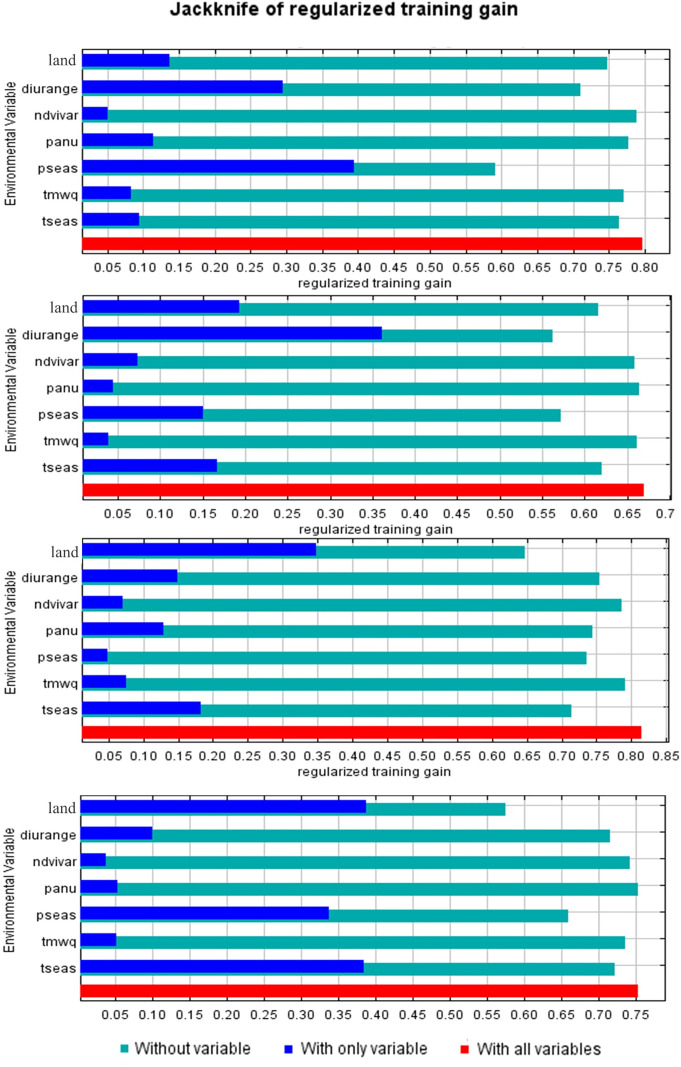
Jackknife test of predictor relevance in environmental suitability models. Predictor codes are shown in S3 Table. From top to bottom: *H. lusitanicum, R.bursa, D. marginatus* and *H. punctata.*

The contribution rates of the predictors included in the models are shown in Table 2. Differences were observedin the top contributing predictors across the models. Land use was consistently among the top three predictors in all four models, and it was the highest contributor for *D. marginatus* (45.6% contribution rate) and *H. punctata* (47.7% contribution rate) model. For *H. lusitanicum*, precipitation seasonality had the highest contribution (42.7%), and it also ranked in the top three for *R. bursa* and *H. punctata*. In the case of *R. bursa*, the annual mean diurnal temperature range showed the highest contribution (47.2%), and it was also one of the main predictors for *H. lusitanicum*. The Jackknife test, which evaluates the explanatory power of each predictor when considered individually, produced consistent results, as the most influential predictor in each test corresponded to the predictor with the highest contribution in the model (Fig 4). These findings suggest that these environmental variables are strongly associated with the predicted distribution of the studied tick species. However, these associations should not be interpreted as direct causal relationships, as they may reflect the effect of other correlated environmental factors that were not explicitly included in the models.

**Table 2:** Relative contribution environmental predictors in tick suitability models.

Predictor	*H. lusitanicum*	*R. bursa*	*D. marginatus*	*H. punctata*
Temperature seasonality	3.4%	8.2%	16.6%	19.2%
Annual mean diurnal range	33.0%	47.2%	9.2%	2.7%
Mean temperature of wettest quarter	4.5%	1.5%	2.1%	2.5%
Annual precipitation	3.1%	1.9%	13.0%	0.0%
Precipitation seasonality	42.7%	15.8%	10.0%	26.2%
Annual NDVI variance	1.5%	2.2%	3.6%	1.9%
Land use	11.7%	23.3%	45.6%	47.7%

**Fig 5:**
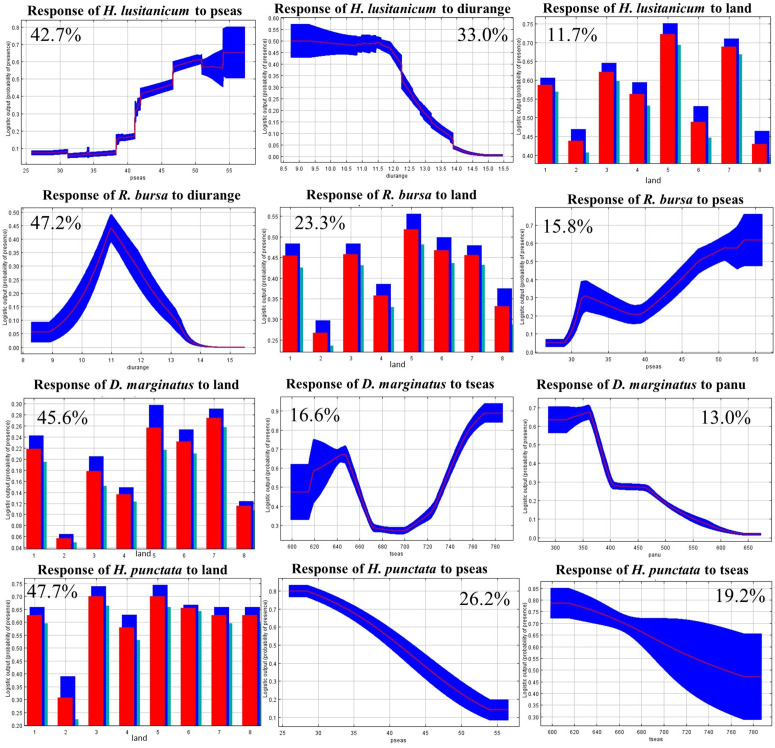
Species response curves for key environmental predictors affecting tick suitability. Species response curves characterizing how the four most important predictors of each model affected the MaxEnt prediction of the four tick studied species suitability model. Predictor abbreviations are shown in S2 Table. From top to bottom: *H. lusitanicum, R. bursa, D. marginatus* and *H. punctata.* The plots show how the logistic prediction changed as each predictor varied while keeping all other predictors at their average sample value. Variable importance in the top left/right corner of each plot. Red lines/bars: mean of the 10 repetitions. Blue area/blue and turquois bars: standard deviation. In the land use (categorical) predictor chart, 1: other land uses; 2: crop; 3: grassland; 4: shrub; 5: deciduous broadleaf forest; 6: evergreen broadleaf forest 7: coniferous forest and 8: woodland.

Species response curves represent the relationship between environmental predictors and the species habitat suitability. Fig 5 illustrates the impact of the top three contributing predictors of each model on the predicted habitat suitability when all other predictors are set to their average value. This allows for the examination of the marginal effect of changing only one predictor. The role of the rest of predictors was not obvious, and the significance of other variables to the optimal model suggests that they are not of high importance in predicting distribution patterns. The response curves of all the predictors included in the models for the different tick species are shown in S6, S7, S8 and S9 Figs. As we eliminated highly correlated predictors, the marginal response curves can be interpreted as the isolated effect of each environmental variable on tick habitat suitability. However, these relationships should still be regarded as associations that may reflect the influence of other, unmeasured correlated variables.. The model for *H. lusitanicum* showed a positive relationship between habitat suitability and precipitation seasonality, within a range of 40–54%. The annual mean diurnal temperature range most favourable to the presence of this tick species was found to be between 9.0 and 11.5°C, after which the habitat suitability sharply decreased. Regarding land use, deciduous broadleaf forest and coniferous forest were the land cover types most strongly associated with habitat suitability for *H. lusitanicum*. In contrast, agricultural areas and woodland were the land cover types with the lowest association to the presence of this species. However, the contribution of this predictor is relatively low (11.7%). The model for *R. bursa* indicated a positive relationship with the annual mean diurnal temperature range between 9 and 11°C, reaching an optimum at this point, after which the relationship becomes negative. Once the diurnal range exceeded 13ºC the habitat suitability drops to below 0.1, suggesting that higher temperature ranges are unfavourable for this species. Deciduous broadleaf forest was the land cover type most strongly associated with predicted habitat suitability, with suitability values exceeding 0.55. Precipitation seasonality was also positively associated with habitat suitability within the range of 40–54%. Therefore, *R. bursa* and *H. lusitanicum* were most likely to be found in natural forested areas with moderate diurnal temperature ranges and high precipitation seasonality, though these associations may be partly driven by other correlated environmental gradients. Areas with extreme diurnal temperature ranges, less natural land uses (such as agriculture), or low precipitation seasonality were less favourable for the species’ presence. For *D. marginatus*, the highest predicted suitability occurred in coniferous forests, followed by deciduous and evergreen broadleaf forests, suggesting an association with forested areas. However, in no case did the habitat suitability exceed 28%, suggesting that *D. marginatus* requires highly specific environmental conditions, even within its most favourable land-use types. Suitability was highest at annual precipitation levels around 360 mm, decreasing at higher values, consistent with adaptation to relatively dry conditions. Temperature seasonality values between 7.5 and 7.8 °C were associated with higher suitability.. Finally, the *H. punctata* suitability model indicated that the habitats with the highest suitability were deciduous broadleaf forests and grasslands, although all natural environments showed relatively high probabilities of presence. Both precipitation seasonality and temperature seasonality exhibited a negative association with the habitat suitability for *H. punctata*. Suitability was highest at precipitation seasonality values between 25% and 30%, declining at higher values.. Regarding temperature seasonality, the habitat suitability was highest within a range of 6.0 to 6.15°C variability, after which it decreased progressively. These findings suggest that *H. punctata* prefers environments with low variability in both precipitation and temperature, indicating that the species may be better adapted to conditions of relatively stable climatic seasonality.

Environmental suitability maps indicated that there were some differences among tick species (Fig 3). *Hyalomma lusitanicum* showed high suitability areas primarily in the west and southwest of CLM. *Rhipicephalus bursa* and *D. marginatus* exhibited a more uniform distribution of suitability across the region. *Haemaphysalis punctata* had higher suitability primarily in the northeast part of CLM.

## Discussion

In the control of many TBDs, prevention by immunisation of the population at risk is not always possible, either because of the lack of preventive tools (vaccines; e.g. CCHF) [61], because their high cost or due to the difficulties in deciding which part of the population to target to be cost-effective [62,63]. Focusing an important part of disease control strategies on preventing tick bites can aid in controlling the problem before it occurs, either by designing strategies to reduce the size of tick populations in areas at high risk of pathogen transmission (e.g. targeted anti-tick vaccines) [64,65], or by designing information campaigns to prevent risky activities in the most vulnerable groups of the population [66,67]. Our study was designed to provide information on where it is most likely to interact with tick species with the highest capacity to transmit pathogens in CLM, informing both animal and human health authorities and the general population where to adopt preventive measures to avoid tick bites and the chances of being infected by TBPs. Preventive measures can be taken on an individual basis, and recommendations for preventing tick bites are publicly available on several websites run by European, national and regional Spanish institutions. Given that models based on the habitat suitability for tick species do not necessarily inform on the risk of interaction, as they are unable to predict population size, combining these models with abundance models provides more valuable epidemiological information. Habitats that are highly favourable for a species may not have been colonised by the species for numerous reasons, or the actual conditions for the species to thrive may not exist because the factors that determine local abundance are not the same as those that determine suitability. Although there are some studies on habitat suitability for different tick species that include CLM, these studies were conducted at the spatial scale of the western Palearctic [68,69], and none of them focused on predicting population abundance. Our results show good spatial agreement in model predictions of environmental suitability and abundance for a highly epidemiologically relevant species in southwestern Europe, *H. lusitanicum*, vector of CCHFV, and agreement for other tick species even though the two models are not fully comparable. These findings provide the basis for decision making on where and how to act in the control of TBDs in CLM.

### Methodological considerations for the interpretation of findings

One aspect that warrants consideration in our study is the potential for bias in the data used to model tick abundance, given that only areas deemed favourable for ticks due to the abundance of wild and domestic ungulates were sampled. The biased selection was based on the dependence of epidemiologically relevant ticks on key hosts such as ungulates [44]. In the absence of key hosts or in intensive ungulate production systems, these ticks are absent or very low in abundance [70] due to the management system and the precise protocolisation of antiparasitic treatments. We started from the assumption that the abundance of these ticks in agricultural landscapes is low and, therefore, that their epidemiological relevance is limited. This potential bias was addressed by calculating the MESS index, thus ensuring a more accurate interpretation of the results. The facts that (1) domestic and wild ungulate abundance indices in surveyed areas presented a wide range [33], (2) that the MESS analysis indicated the areas where model predictions were less reliable in terms of environmental predictors, and (3) that the validation of the models presented correlation coefficients between predicted and observed values around 0.6 in the four models, indicate that the predictive capacity of the models accurately identified areas of medium-high abundance of these ticks [5] and of highest risk of interaction with ticks. The high concordance between the habitat suitability maps and the abundance maps for some of the tick species included in this study also supports this perception. However, we recognise that these models may over/underestimate the abundance of these ticks in particular areas of the study region, so more comprehensive sampling data will allow us to improve their predictive value and the accuracy of the estimates in the future.

In spatial studies, the scale of analysis is crucial, as the influence of environmental factors on ecological processes may vary according to the spatial scale used [71]. Thus, using finer spatial scales can result in more informative maps [72]. At the spatial scale at which we constructed abundance models, host availability may be an important parameter in shaping tick population size along with other biotic and abiotic parameters [5,11]. However, similar tick abundance modelling approaches at similar spatial scales with *Ixodes ricinus* in northern Spain [5] found a lower role of host abundance on the spatial patterns of tick abundance than that of other biotic and of biotic factors. Only at very small spatial scales, the role of key host abundance in shaping tick abundance patters is clear [11]. We do not know the host range for some of the tick species in this study, mainly of immature stages (larva and nymph) such as *D. marginatus* and *H. punctata*, and we have limited information for *H. lusitanicum* [29]. The regional government of CLM records annual hunting statistics, but currently only environmental suitability models for wild ungulates are available [33] and there are no records of abundance of non-game wild species in the region.

Another aspect to consider before interpreting the results is that abundance and suitability are not necessarily correlated. While the initial assumption might be that more suitable areas will host a greater abundance of ticks, this is not always the case. The factors influencing suitability and abundance can differ, and abundance is also the result of demographic processes such as reproduction, survival, and growth [73,74].

### Tick spatial distribution and tick-borne pathogen transmission risk

One of the main arguments for the development of this study was the emergence of CCHF in CLM and the potential emergence/re-emergence of other pathogens of medical and veterinary relevance transmitted by *H. lusitanicum* [22]. Thus, we started from the initial hypothesis that the spatial distribution of *H. lusitanicum* in CLM would be greater than known and that it would be the predominant species in the region, as previous studies in central Spain seemed to indicate [12]. However, we observed significant heterogeneity in tick populations across different locations in CLM. The highest number of *H. lusitanicum* was captured in western CLM, which is consistent with several studies carried out in this part of the region, both for ticks collected in the vegetation [12,75] and on animals [29]. However, *H. lusitanicum* was only the predominant tick on vegetation in two of the surveyed sites in southwestern CLM, but not in the rest of sites in which it was found present, contradicting the initial hypothesis. Its distribution range was, however, found to be wider than initially expected, being it present in almost the entire region based both on long-term records of ticks and on the questing tick survey (Fig 3). Uncertainty in the spatial projection of the abundance model for *H. lusitanicum* was low in western CLM, where the suitability model identified the highest habitat suitability at medium to high abundance. The number of *H. lusitanicum* collected in vegetation surveys in eastern CLM was very low, only one adult in region A5. This is consistent with the low environmental suitability for the species in this part of the region. However, the review of animal records indicates the presence of the species in eastern CLM, showing that the species could colonize marginally favourable niches in which to proliferate in the future under changes in environmental conditions.

There are suspicions that species of the genus *Rhipicephalus* [76] and *D. marginatus* [77] may be involved in the maintenance and transmission of CCHFV, but we still lack the appropriate experimental prove. However, the spatial distribution pattern of *H. lusitanicum* in CLM almost perfectly shapes the predicted spatial pattern of CCHFV [17,18,78]. This suggests a limited role of non*-Hyalomma* ticks in CCHFV maintenance when *Hyalomma* ticks are absent or at low abundance. However, the potential spatial spread of *Hyalomma* ticks in CLM in the future should not be disregarded as a relevant modulator of changes in the current distribution of the virus.

Many of the other TBPs previously detected in CLM [22,23] are maintained and transmitted by non-*Hyalomma* ticks [79–81]. *Rhipicephalus bursa* and *D. marginatus* may be important vectors for some species of the genera *Anaplasma*, *Rickettsia* and *Ehrlichia* [82–84], and *H. punctata* can maintain pathogens of the genera *Rickettsia*, *Babesia* and *Theileria* [85,86]. *Rhipicephalus bursa* predominated over other questing tick species at seven of 20 sampling points, indicating a high ecological plasticity that perhaps, in part, is associated to its two-host life strategy [87] and its dependence on wild ungulates [11] for the maintenance of some (if not all) of its developmental stages [37]. The low uncertainty in the predictions of the areas of greatest abundance of *R. bursa*, with the exception of some squares in the northeast of the region, support the environmental versatility of the species, which also has even been described with relevant presence in eastern, central and northern Spain [5,30,88]. *Dermacentor marginatus* exhibited a fairly homogeneous distribution in the different sampling areas, similar to *R. bursa,* perhaps taking advantage of the wide distribution and abundance of the Eurasian wild boar in CLM [33] which is a relevant host for *D. marginatus* adults [11,30]. However, the congruence of the predicted environmental suitability and abundance patterns was the lowest among the four tick species studied. Indeed, the predictive capacity of the abundance model for *D. marginatus* was not good as indicated by a wide confidence interval in the Pearson correlation test and an over/underestimation of abundance. It appears that *D. marginatus* abundance is less associated with abiotic and habitat factors as observed in previous studies [89–91], with host abundance perhaps being the best predictor [92]. *Haemaphysalis punctata* was most frequently captured in the northeast of CLM. The predicted environmental suitability and abundance patterns of *H. punctata* were also similar, with models suggesting higher environmental suitability and abundance in the northeast of CLM. In this case, the correlation plots indicate that the abundance model is effective in differentiating between areas with high and low abundance. We also identified some highly suitable areas in the western part of the region, where we have occasionally recorded *H. punctata*. However, these were areas with high uncertainty, so caution is needed when interpreting abundance results.

### Local traits shaping ixodid tick environmental suitability and abundance

In examining the underlying factors that contribute to these predictions of abundance and environmental suitability, it becomes evident that there are both intra- and inter-specific similarities and differences.

### Hyalomma lusitanicum

For *H. lusitanicum*, the most relevant climatic predictors of environmental suitability were also selected in the abundance model. Furthermore, both models showed similar effects of the selected predictors; precipitation seasonality positively correlated with *H. lusitanicum* presence and abundance, whereas the annual mean diurnal range exhibited a negative correlation with both parameters. The response curves of the suitability model suggested that *H. lusitanicum* favours habitats with moderate to high precipitation seasonality and lower daily temperature fluctuations. The association of precipitation seasonality with *H. lusitanicum* presence and abundance is consistent with previous studies that identified an increased risk of exposure to CCHFV with increasing seasonality in annual rainfall patterns [18]. CCHFV exposure risk is strongly associated to the local abundance of *H. lusitanicum* ticks [11]. The abundance of *H. lusitanicum* was also influenced by other climatic factors, including annual accumulated precipitation and temperature seasonality. The former had a negative effect, while the latter had a positive one instead. Neither of these factors had a relevant impact on the habitat suitability for *H. lusitanicum*, but according to response curves, the effect of these two factors on suitability was complex and non-lineal. The suitability model suggests that *H. lusitanicum* selects areas where annual rainfall is around or above 400 mm; under this precipitation threshold, the habitat becomes marginally suitable. Therefore, *H. lusitanicum* seems to thrive in habitats with some degree of soil moisture while it does not colonize very dry environments. A preference for humid environments within meso-Mediterranean climates was also observed in previous studies where NDVI - a factor related to water stress - was found to be highly associated to *H. lusitanicum* abundance [11] and CCHFV exposure risk [17] at smaller spatial scales. The negative relationship of the annual accumulated precipitation and *H. lusitanicum* abundance in our study may be related to the spatial scale of the analysis. The northeast of CLM is the rainiest and cooler area compared to the rest of the region. As we previously mentioned, the interpretation of individual predictor effects must be approached with caution. In this area, the most limiting climatic factor might be temperature, as *H. lusitanicum* seems to thrive in warmer climates. This aligns with previous studies that observed that *H. lusitanicum* is adapted to the warm conditions of the meso-Mediterranean climate [12,75].

Regarding land use, we observed both similarities and differences between abundance and suitability models. While the abundance model identified a negative relationship between *H. lusitanicum* and shrubland, this was not observed in the suitability model. This may be related to the type of sampling, as it is well-established that blanket dragging is not equally effective across all habitat types, and this may be what the model is capturing [35]. This was also reflected in the *R. bursa* models. On the other hand, the data indicated that in both models, coniferous forests exhibited a positive correlation with *H. lusitanicum*. Lastly, the most favourable habitat for *H. lusitanicum* appeared to be deciduous broadleaf forest, but this land use did not seem to affect the abundance of this species. In this study, *H. lusitanicum* was captured across a wide range of habitats, including both mixed and Mediterranean forests. These results differ from previous studies in Spain where *H. lusitanicum* abundance was negatively associated with pine forests [11] or wooded areas in general [16]. Furthermore, in CLM, it was found that evergreen forests harbour the highest abundance of *H. lusitanicum* [12,75]. The discrepancies in habitat preferences observed across studies may be attributed to the influence of additional interacting factors within the habitat or to the efficiency of the sampling method in estimating tick abundance. These findings highlight the relevance of the spatial scale of the and the potential influence of local traits on the presence/abundance of a tick species.

### Rhipicephalus bursa

The influence of local environmental traits on *R. bursa* presence and abundance appears somewhat more complex than in *H. lusitanicum*. While the climatic predictors mostly agree in both models (though the suitability patterns are somewhat more intricate), habitat factors selected by suitability and abundance models differ.

Similarly to *H. lusitanicum, R. bursa* is adapted to warm and dry climates and is very common in the Mediterranean region [25,93]. Thus, both species exhibit comparable selection patterns regarding local precipitation seasonality and daily temperature fluctuations. However, annual precipitation demonstrated a positive correlation with *R. bursa* abundance in contrast to the effect observed on *H. lusitanicum* abundance. Studies in North Africa suggest that, despite its adaptation to arid climates, *R. bursa* is restricted to the least arid regions within arid or semi-arid areas [94]. The detailed examination of the effect of land use factors on *R. bursa* presence and abundance reveals important differences. Deciduous forests were identified as the potentially preferentially selected habitat of *R. bursa*. However, deciduous forests negatively correlated with *R. bursa* abundance. Probably, additional biotic/abiotic factors are driving the relationship between the presence and abundance of *R. bursa* and deciduous forests. One notable observation is that several land uses exhibit a relatively similar suitability for *R. bursa* presence (~0.45), suggesting that we are dealing with a more generalist species in terms of habitat selection. We mainly found a preference for forests of *R. bursa*, but also for grasslands, which partially agrees with previous results [16].

### Dermacentor marginatus

While the spatial predicted distribution of *D. marginatus* in abundance and environmental suitability models did not exhibit a strong correlation, an examination of the influencing factors reveals that both abundance and suitability were associated to land use. Similarly to *H. lusitanicum*, coniferous and deciduous forests are probably the most suitable habitats for *D. marginatus*. These predictors were also positively correlated with *D. marginatus* abundance. This suggests that *D. marginatus* preferentially selects forest habitats within CLM, as shown for southern Italy [91]. However, in other areas, it selects a diversity of habitats [91,92]. These observations are perhaps related to local variation in habitat preference of its hosts, particularly the Eurasian wild boar, which is primarily a forest-dwelling species but can inhabit almost any type of habitat [95,96]. This reinforces the necessity of considering the scale and location of the study, and the inadvisability of extrapolating the results of the models for *D. marginatus* suitability and abundance with precision to CLM, which is also in agreement with the results of the MESS analysis for *D. marginatus* models. The climatic predictors with a higher influence on the environmental suitability for *D. marginatus* and its abundance were precipitation and temperature. Similarly to *H. lusitanicum* and *R. bursa*, *D. marginatus* has evolved to thrive in arid climates [16]. However, the inverse correlation between the mean temperature of the wettest season of the year and *D. marginatus* abundance suggests that it can adapt to colder areas better than *H. lusitanicum* and *R. bursa*. This adaptation to slightly colder temperatures may drive its seasonal activity peak that is observed in autumn. Therefore, although *D. marginatus* may be adapted to semi-arid lands, it may be not well adapted to hot areas. Currently favourable areas of CLM may become marginally favourable with increasing temperatures linked to global warming. This may have positive consequences if it results in a decreased transmission risk of spotted-fever group *Rickettsia* spp. for which *D. marginatus* is a relevant vector [23].

### Haemaphysalis punctata

*Haemaphysalis punctata* seems to exhibit remarkable ecological adaptability, tolerating a wide range of climatic conditions and diverse habitats [85]. This agrees with the findings of our suitability model, where the majority of land uses (with the exception of cultivated areas) exhibited a comparable and elevated habitat suitability for *H. punctata* (>0.55). In contrast, this was not reflected in the abundance model as it did not identify any habitat predictor as relevant. It appears that *H. punctata* abundance may be mainly associated to climatic predictors or, probably, to host presence/abundance. *Haemaphysalis punctata* is commonly known as the red sheep tick, as it appears to have a preference for sheep [85], as well as for mouflons (wild sheep) in its adult stage [97]. This suggests that these vertebrates may play a key role in *H. punctata* presence and abundance where environmental conditions are favourable for the species to thrive. Birds also seem to be important hosts for *H. punctata* immatures, being the most prevalent tick species on fall migrants in Mediterranean areas [98]. The observed influence of climatic factors indicates that *H. punctata* is associated with colder and wetter climates than the other three ticks studied [25]. It is indeed one of the most common species in northern Spain [16]. However, in northern Spain, *H. punctata* is more abundant in drier Mediterranean climate influenced regions than in more humid Atlantic climatic areas [5]. This was also reflected in our study, which revealed a negative, albeit not statistically significant, correlation between mean temperature of the wettest quarter and both abundance and habitat suitability. Also, we observed a negative correlation between annual precipitation and *H. punctata* abundance. Most captures and *H. punctata* presence records were concentrated in the rainiest and coolest area of CLM, specifically in the northeast of the region. While this species seems to select more humid areas in Mediterranean regions, it appears to avoid extremely humid areas in northern Spain [5]. This agrees with the observed relationship between precipitation seasonality and *H. punctata* abundance and environmental suitability. Additionally, the negative relationship of temperature seasonality on the environmental suitability for *H. punctata* suggests a preference for areas with more stable temperatures.

## Conclusions

The present study reveals that tick populations across CLM are characterised by considerable heterogeneity. We identified three species of ticks (*H. lusitanicum, D. marginatus,* and *R. bursa*) which are well adapted to the dry and warm meso-Mediterranean climates, with *H. lusitanicum* showing a more restricted distribution to the west/southwest of the region. In contrast, *H. punctata* was observed in the northeast of the region, where the climate is characterised by higher humidity and lower temperatures. No significant differences were observed in habitat preferences, although some species, such as *H. punctata*, appeared to exhibit a more generalist behaviour than others, like *H. lusitanicum*. In general, it can be concluded that the use of climatic and land use predictors is an effective proxy for understanding the abundance and distribution of these tick species in CLM, except for *D. marginatus*, whose abundance appears to be highly influenced by other factors. Given the relevance of these tick species to human and animal health, the information provided on their distribution and abundance in the region can be a crucial milestone for health authorities in implementing measures to prevent TBDs by adopting strategies to prevent tick bites where and when these could be more efficient.

## Supporting information

S1 Table Results of the questing tick surveys. Hl: *Hyalomma lusitanicum*; Hm: *Hyalomma marginatum;* Rb: *Rhipicephalus bursa*; Rs: *Rhipicephalus sanguineus* sensu lato.; Ir: *Ixodes ricinus;* Dm: *Dermacentor marginatus;* Hp: *Haemaphysalis punctata.*_a_: adult; _n_: nymph; _l_: larvae. The species targeted in this study are shown in bold type letter case.
(XLSX)

S2 Table Set of explanatory predictors considered as potential tick abundance determinants. Predictors that were used for model building after the descriptive analysis are marked in bold italic letter case type. Values represent the average and range of predictor variables within a 1-km buffer around the transects where adult ticks of each species were collected. Hl: *Hyalomma lusitanicum*; Rb: *Rhipicephalus bursa*; Dm: *Dermacentor marginatus;* Hp: *Haemaphysalis punctata.*
(XLSX)

S3 Table Environmental predictors considered for tick habitat suitability predictive modelling. Predictors that were used for model building after the descriptive analysis are marked in bold italic letter case type. Values represent the average and range of predictor variables extracted from 1 × 1 km raster cells containing occurrence records for each tick species. Hl: *Hyalomma lusitanicum*; Rb: *Rhipicephalus bursa*; Dm: *Dermacentor marginatus;* Hp: *Haemaphysalis punctata.*
(XLSX)

S4 Table Estimated questing density. Estimated questing density (ticks/hectare) of the adult tick species studied in the 20 sampling sites of the study. Hl: *Hyalomma lusitanicum;* Rb: *Rhipicephalus bursa*; Dm: *Dermacentor marginatus;* Hp: *Haemaphysalis punctata.*
(XLSX)

S5 Table Number of adult ticks collected. Summary of the accumulated number of adult ticks collected in the 20 sampling sites of study across the survey period and the estimated questing density (ticks/hectare) in each season of the year. Hl: *Hyalomma lusitanicum;* Rb: *Rhipicephalus bursa*; Dm: *Dermacentor marginatus;* Hp: *Haemaphysalis punctata.*
(XLSX)

S1 Fig Correlation matrix. Mutual correlation (correlation matrix) among the environmental predictors considered as potentially influencing adult tick presence and abundance (See predictors in S2 Table). The colour blue is indicative of a positive correlation, whereas the colour red is indicative of a negative correlation; the Pearson correlation coefficient (r) is higher as darker is the blue or red colour. The strength of the correlation in absolute value is also shown with increasing size of inner colour squares; a large size indicates a strong correlation, while a smaller one indicates a weaker correlation. The predictors having correlation coefficients r > |0.7| are grouped together according to hierarchical cluster analysis.
(TIF)

S2 Fig Number of ticks captured per season of the year. a) *Hyalomma lusitanicum,* b) *Rhipicephalus bursa,* c) *Dermacentor marginatus,* d) *Haemaphysalis punctata.* (TIF)

S3 Fig QQPlots of residuals for tick abundance models. QQPlots showing the results of the analysis of the residuals of the tick abundance models. a) *Hyalomma lusitanicum,* b) *Rhipicephalus bursa,* c) *Dermacentor marginatus,* d) *Haemaphysalis punctata.* Plot was generated using the ‘PlotQQunif’ function to the object created after applying the ‘simulateResiduals’ function, both functions in the ‘DHARMa’ package of R.
(TIF)

S4 Fig Calibration plots of tick abundance models. Calibration plots of the abundance models parameterized with 70% of the data and validated with the remaining 30%. The X-axis represents the model predicted questing abundance or relative abundance (No. of questing adult ticks per transect corrected by transect length). The Pearson correlation coefficient and the 95% confidence interval are displayed in the top-left part of each chart. a) *Hyalomma lusitanicum,* b) *Rhipicephalus bursa,* c) *Dermacentor marginatus,* d) *Haemaphysalis punctata.*
(TIF)

S5 Fig Omission and ROC curves for environmental suitability models. Omission versus predicted data (left charts) and receiver operating characteristic curve with the area under curve (AUC; right charts), of environmental suitability models. *Hyalomma lusitanicum* (a, b); *Rhipicephalus bursa* (c, d); *Dermacentor marginatus* (e, f); *Haemaphysalis punctata* (g, h).
(TIF)

S6 Fig Species response curves for *Hyalomma lusitanicum* suitability model. Species response curves characterizing how each predictor influenced the MaxEnt prediction in *Hyalomma lusitanicum* suitability model. Left charts show how the logistic prediction changed as each predictor varied while keeping all other predictors at their average sample value. Right charts show MaxEnt models created using only the corresponding variable. Red lines/bars: mean of the 10 repetitions. Blue area/blue and turquois bars: standard deviation. In the land (categorical predictor) chart (bottom), 1: other land uses; 2: crop; 3: grassland; 4: shrub; 5: deciduous broadleaf forest; 6: evergreen broadleaf forest 7: coniferous forest and 8: woodland.
(TIF)

S7 Fig Species response curves for *Rhipicephalus bursa* suitability model. Species response curves characterizing how each predictor influenced the MaxEnt prediction in *Rhipicephalus bursa* suitability model. Left charts show how the logistic prediction changed as each predictor varied while keeping all other predictors at their average sample value. Right charts show MaxEnt models created using only the corresponding variable. Red lines/bars: mean of the 10 repetitions. Blue area/blue and turquois bars: standard deviation. In the land (categorical predictor) chart (bottom), 1: other land uses; 2: crop; 3: grassland; 4: shrub; 5: deciduous broadleaf forest; 6: evergreen broadleaf forest 7: coniferous forest and 8: woodland.
(TIF)

S8 Fig Species response curves for *Dermacentor marginatus* suitability model. Species response curves characterizing how each predictor influenced the MaxEnt prediction in *Dermacentor marginatus* suitability model. Left charts show how the logistic prediction changed as each predictor varied while keeping all other predictors at their average sample value. Right charts show MaxEnt models created using only the corresponding variable. Red lines/bars: mean of the 10 repetitions. Blue area/blue and turquois bars: standard deviation. In the land (categorical predictor) chart (bottom), 1: other land uses; 2: crop; 3: grassland; 4: shrub; 5: deciduous broadleaf forest; 6: evergreen broadleaf forest 7: coniferous forest and 8: woodland.
(TIF)

S9 Fig Species response curves for *Haemaphysalis punctata* suitability model. Species response curves characterizing how each predictor influencing the MaxEnt prediction in *Haemaphysalis punctata* suitability model. Left charts show how the logistic prediction changed as each predictor varied while keeping all other predictors at their average sample value. Right charts show MaxEnt models created using only the corresponding variable. Red lines/bars: mean of the 10 repetitions. Blue area/blue and turquois bars: standard deviation. In the land (categorical predictor) chart (bottom), 1: other land uses; 2: crop; 3: grassland; 4: shrub; 5: deciduous broadleaf forest; 6: evergreen broadleaf forest 7: coniferous forest and 8: woodland.
(TIF)
