# Supplementary figures and images for: Modelling tick abundance and environmental suitability in meso-Mediterranean landscapes for the prevention of tick-borne diseases
Source: PLoS Negl Trop Dis. 2025 Nov 17;19(11):e0013741. doi: 10.1371/journal.pntd.0013741 (PMC12671887; doi:10.1371/journal.pntd.0013741)

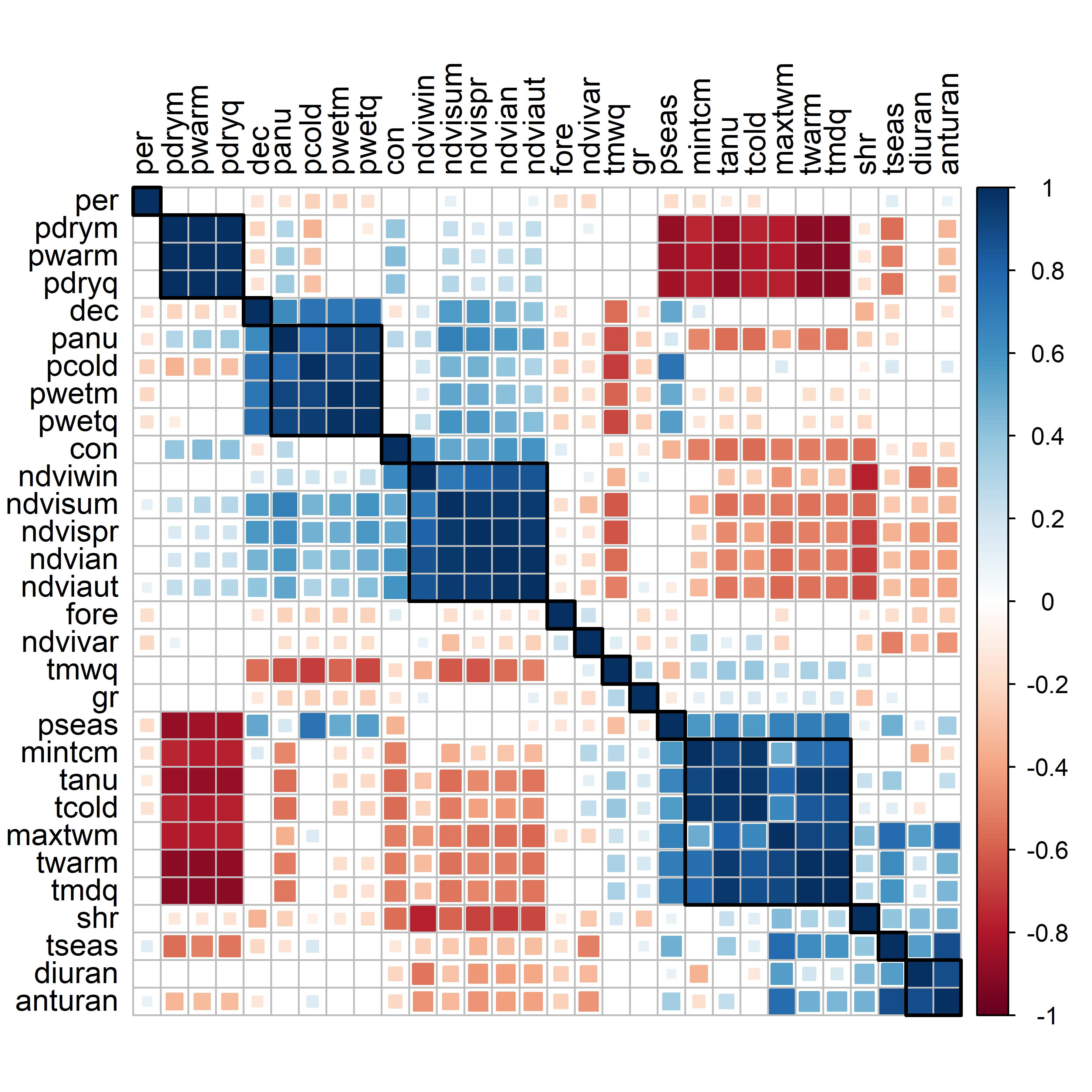

Supplement: S1 Fig — Mutual correlation (correlation matrix) among the environmental predictors considered as potentially influencing adult tick presence and abundance (See predictors in S2 Table). The colour blue is indicative of a positive correlation, whereas the colour red is indicative of a negative correlation; the Pearson correlation coefficient (r) is higher as darker is the blue or red colour. The strength of the correlation in absolute value is also shown with increasing size of inner colour squares; a large size indicates a strong correlation, while a smaller one indicates a weaker correlation. The predictors having correlation coefficients r > |0.7| are grouped together according to hierarchical cluster analysis. (TIF) [file pntd.0013741.s006.tif]

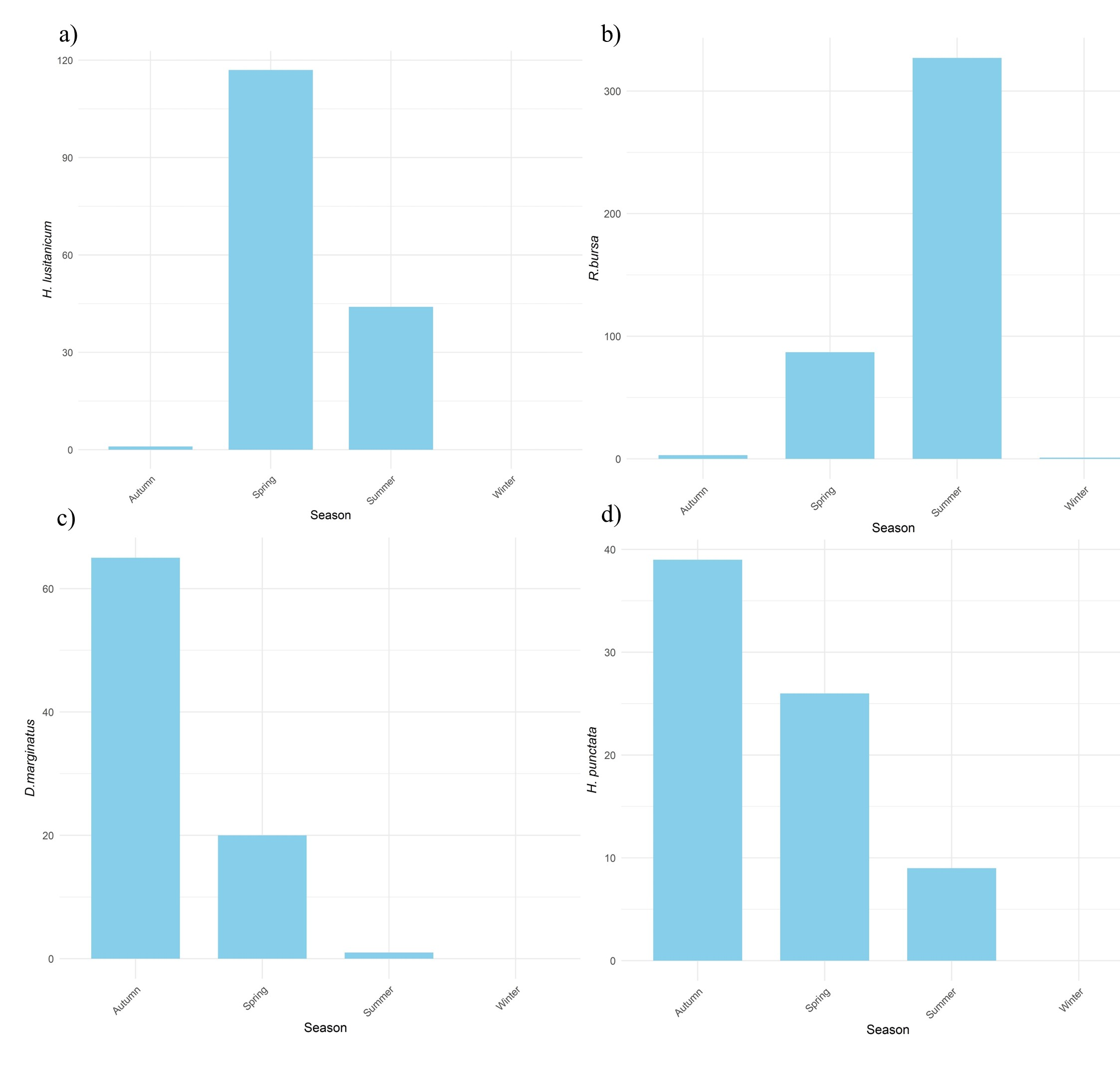

Supplement: S2 Fig — (TIF) [file pntd.0013741.s007.tif]

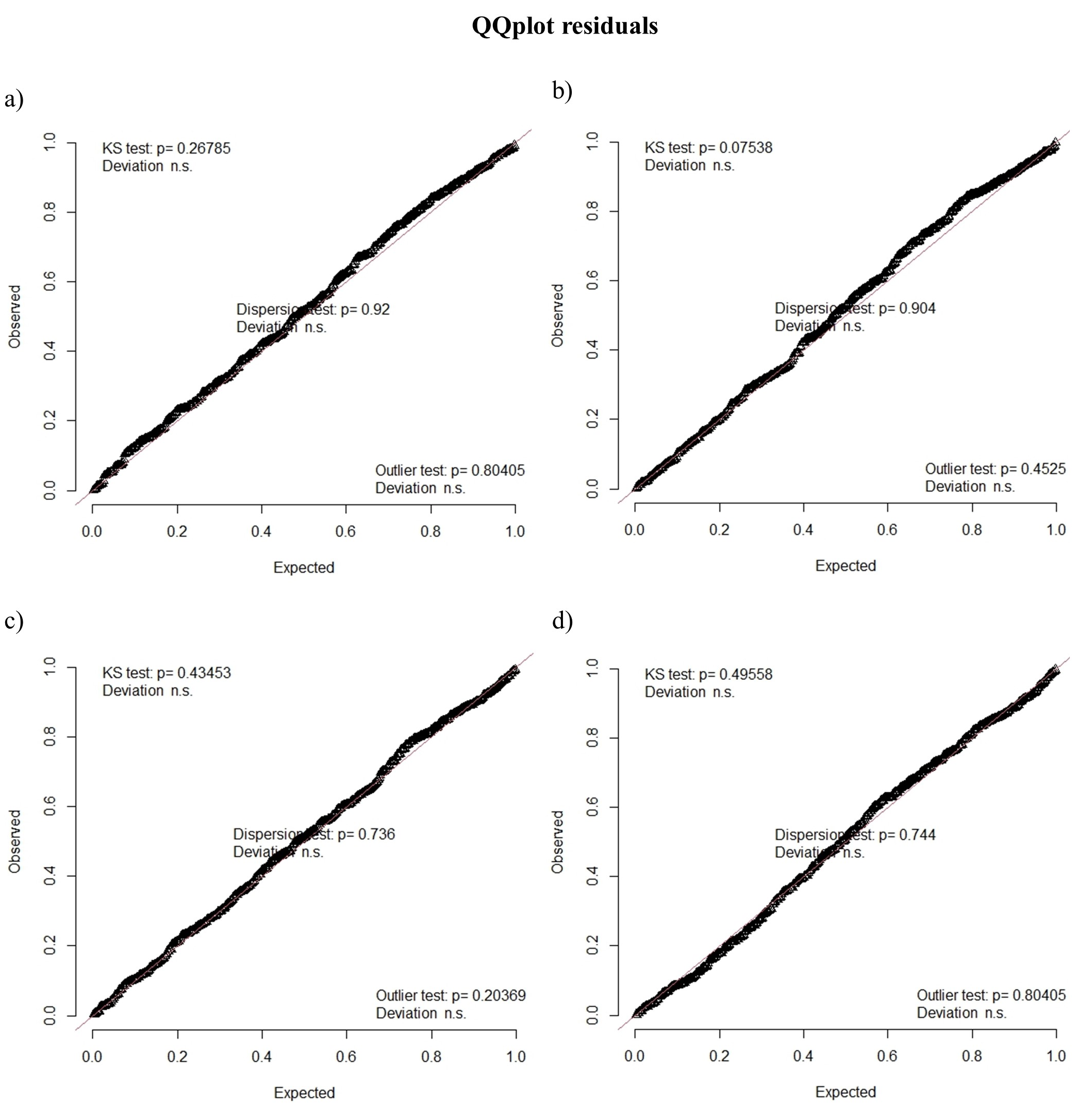

Supplement: S3 Fig — QQPlots showing the results of the analysis of the residuals of the tick abundance models. a) Hyalomma lusitanicum, b) Rhipicephalus bursa, c) Dermacentor marginatus, d) Haemaphysalis punctata. Plot was generated using the ‘PlotQQunif’ function to the object created after applying the ‘simulateResiduals’ function, both functions in the ‘DHARMa’ package of R. (TIF) [file pntd.0013741.s008.tif]

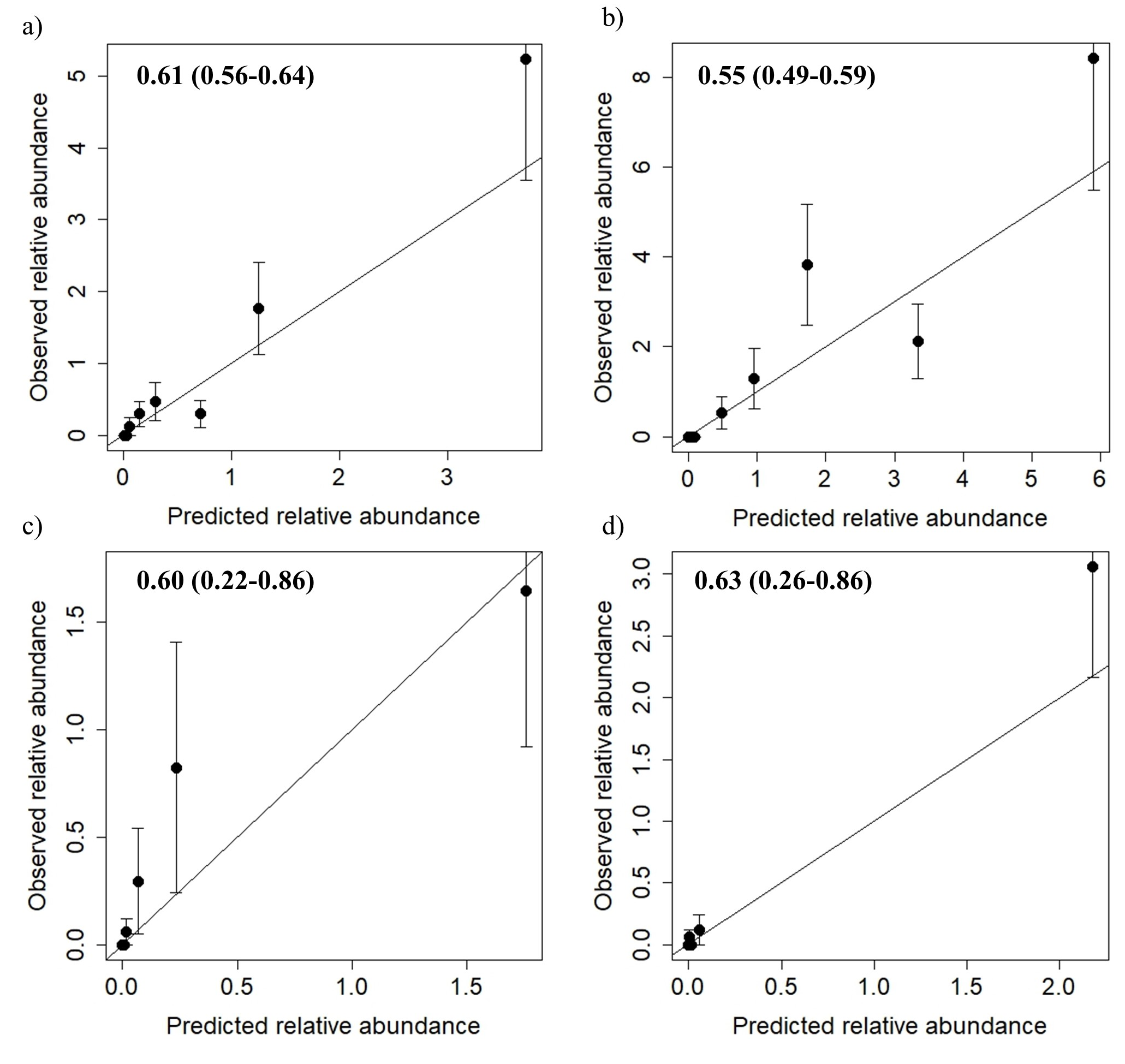

Supplement: S4 Fig — Calibration plots of the abundance models parameterized with 70% of the data and validated with the remaining 30%. The X-axis represents the model predicted questing abundance or relative abundance (No. of questing adult ticks per transect corrected by transect length). The Pearson correlation coefficient and the 95% confidence interval are displayed in the top-left part of each chart. a) Hyalomma lusitanicum, b) Rhipicephalus bursa, c) Dermacentor marginatus, d) Haemaphysalis punctata. (TIF) [file pntd.0013741.s009.tif]

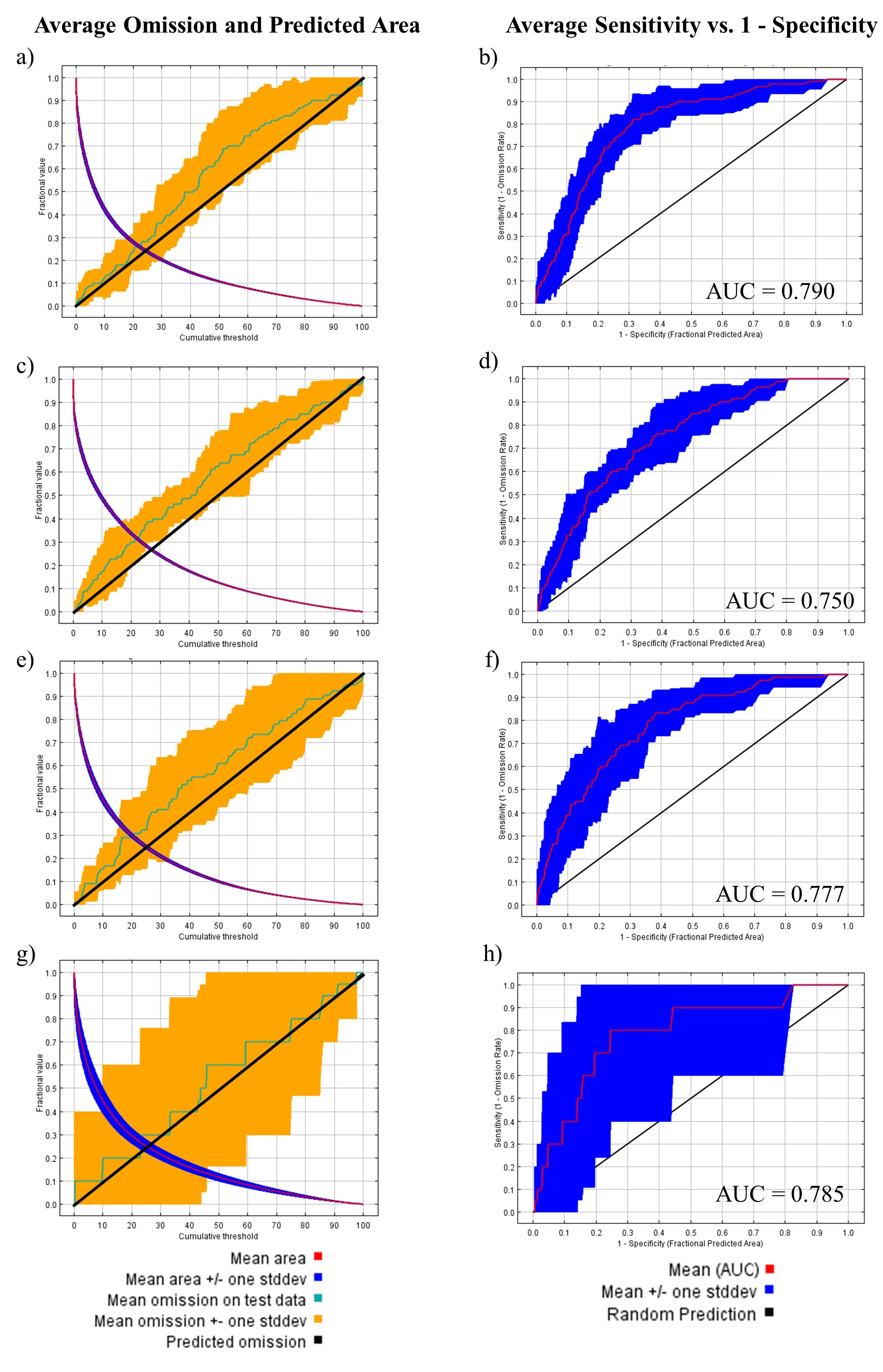

Supplement: S5 Fig — Omission versus predicted data (left charts) and receiver operating characteristic curve with the area under curve (AUC; right charts), of environmental suitability models. Hyalomma lusitanicum (a, b); Rhipicephalus bursa (c, d); Dermacentor marginatus (e, f); Haemaphysalis punctata (g, h). (TIF) [file pntd.0013741.s010.tif]

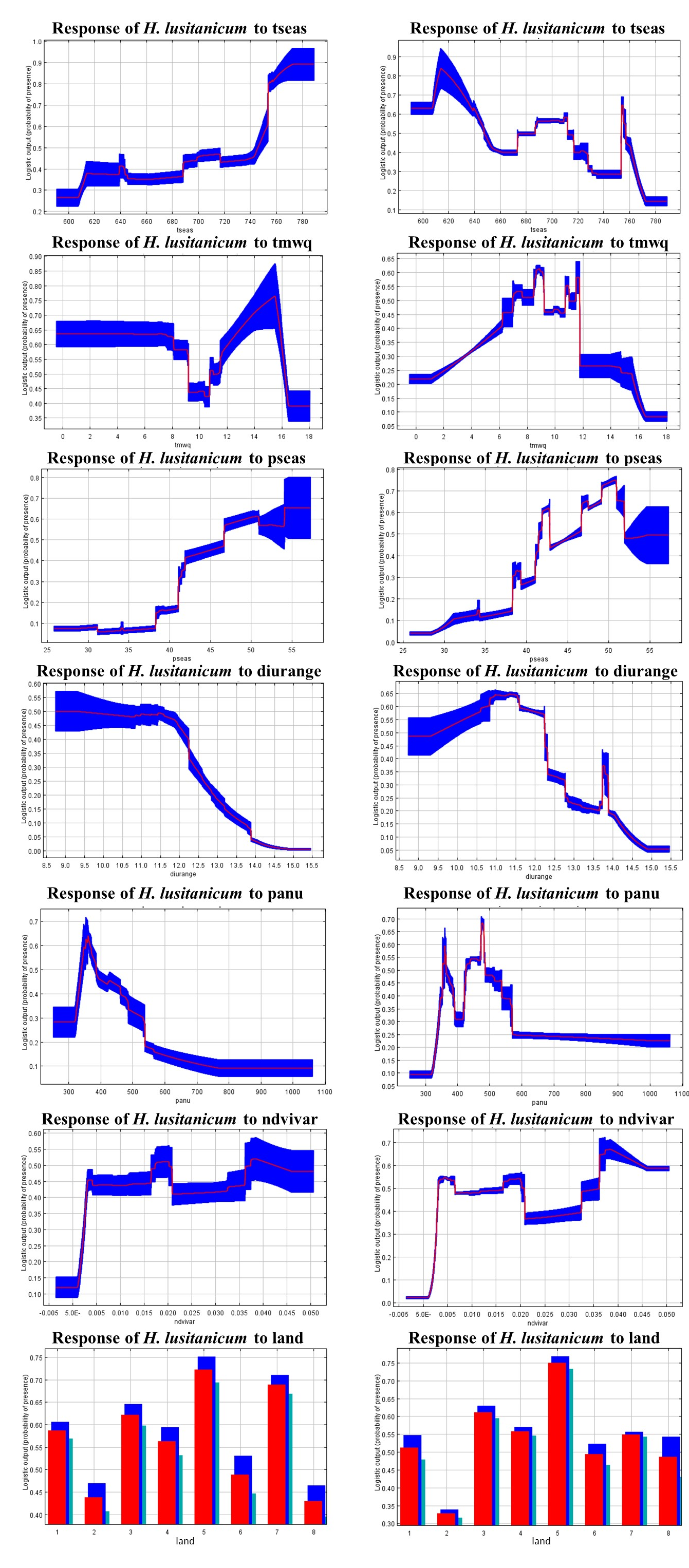

Supplement: S6 Fig — Species response curves characterizing how each predictor influenced the MaxEnt prediction in Hyalomma lusitanicum suitability model. Left charts show how the logistic prediction changed as each predictor varied while keeping all other predictors at their average sample value. Right charts show MaxEnt models created using only the corresponding variable. Red lines/bars: mean of the 10 repetitions. Blue area/blue and turquois bars: standard deviation. In the land (categorical predictor) chart (bottom), 1: other land uses; 2: crop; 3: grassland; 4: shrub; 5: deciduous broadleaf forest; 6: evergreen broadleaf forest 7: coniferous forest and 8: woodland. (TIF) [file pntd.0013741.s011.tif]

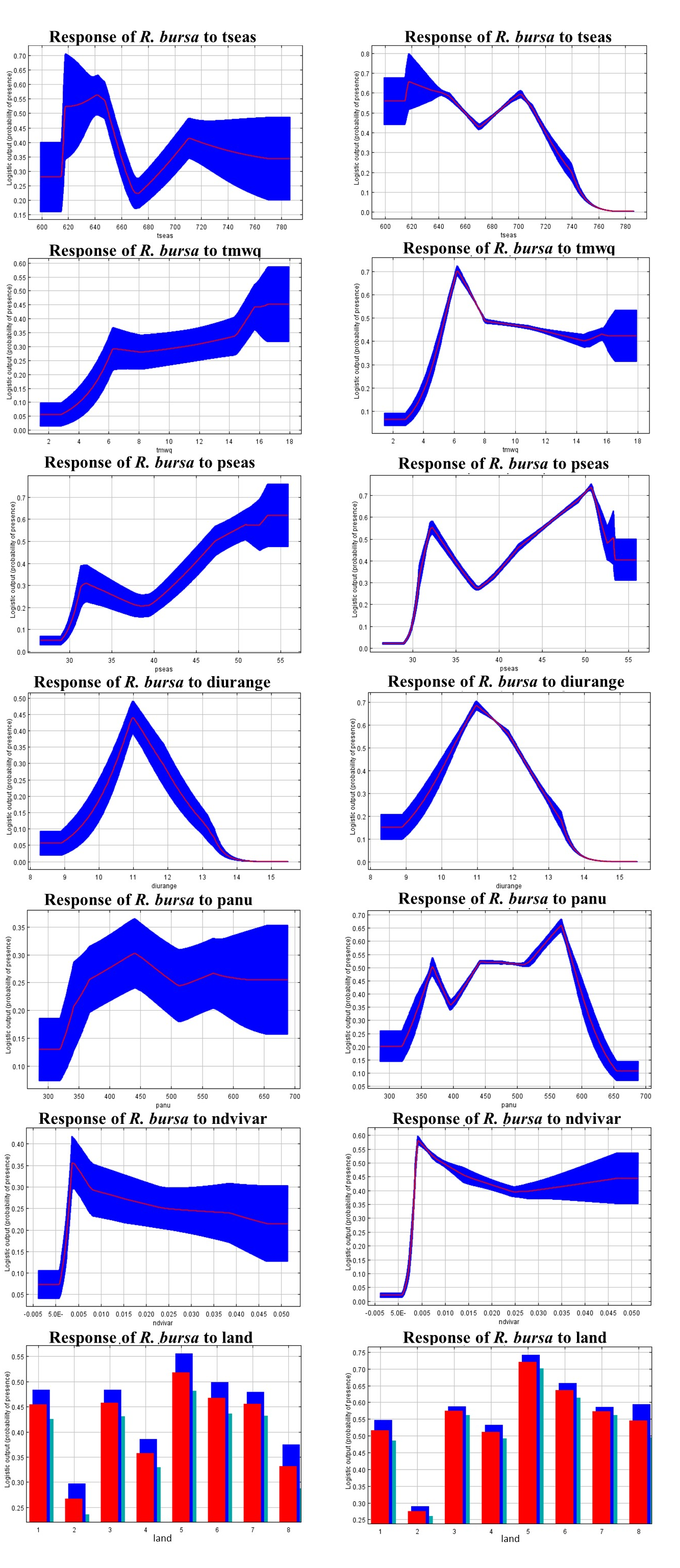

Supplement: S7 Fig — Species response curves characterizing how each predictor influenced the MaxEnt prediction in Rhipicephalus bursa suitability model. Left charts show how the logistic prediction changed as each predictor varied while keeping all other predictors at their average sample value. Right charts show MaxEnt models created using only the corresponding variable. Red lines/bars: mean of the 10 repetitions. Blue area/blue and turquois bars: standard deviation. In the land (categorical predictor) chart (bottom), 1: other land uses; 2: crop; 3: grassland; 4: shrub; 5: deciduous broadleaf forest; 6: evergreen broadleaf forest 7: coniferous forest and 8: woodland. (TIF) [file pntd.0013741.s012.tif]

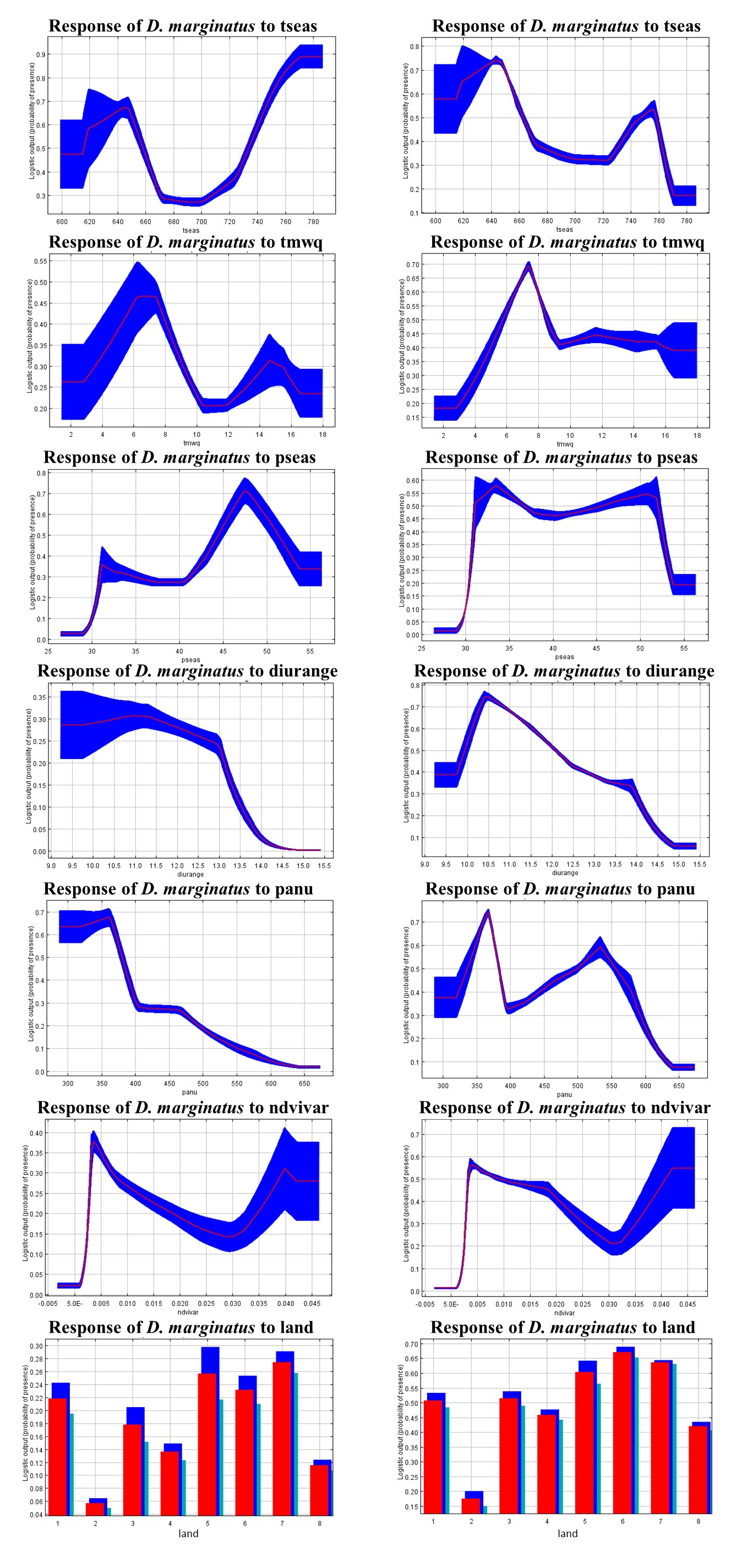

Supplement: S8 Fig — Species response curves characterizing how each predictor influenced the MaxEnt prediction in Dermacentor marginatus suitability model. Left charts show how the logistic prediction changed as each predictor varied while keeping all other predictors at their average sample value. Right charts show MaxEnt models created using only the corresponding variable. Red lines/bars: mean of the 10 repetitions. Blue area/blue and turquois bars: standard deviation. In the land (categorical predictor) chart (bottom), 1: other land uses; 2: crop; 3: grassland; 4: shrub; 5: deciduous broadleaf forest; 6: evergreen broadleaf forest 7: coniferous forest and 8: woodland. (TIF) [file pntd.0013741.s013.tif]

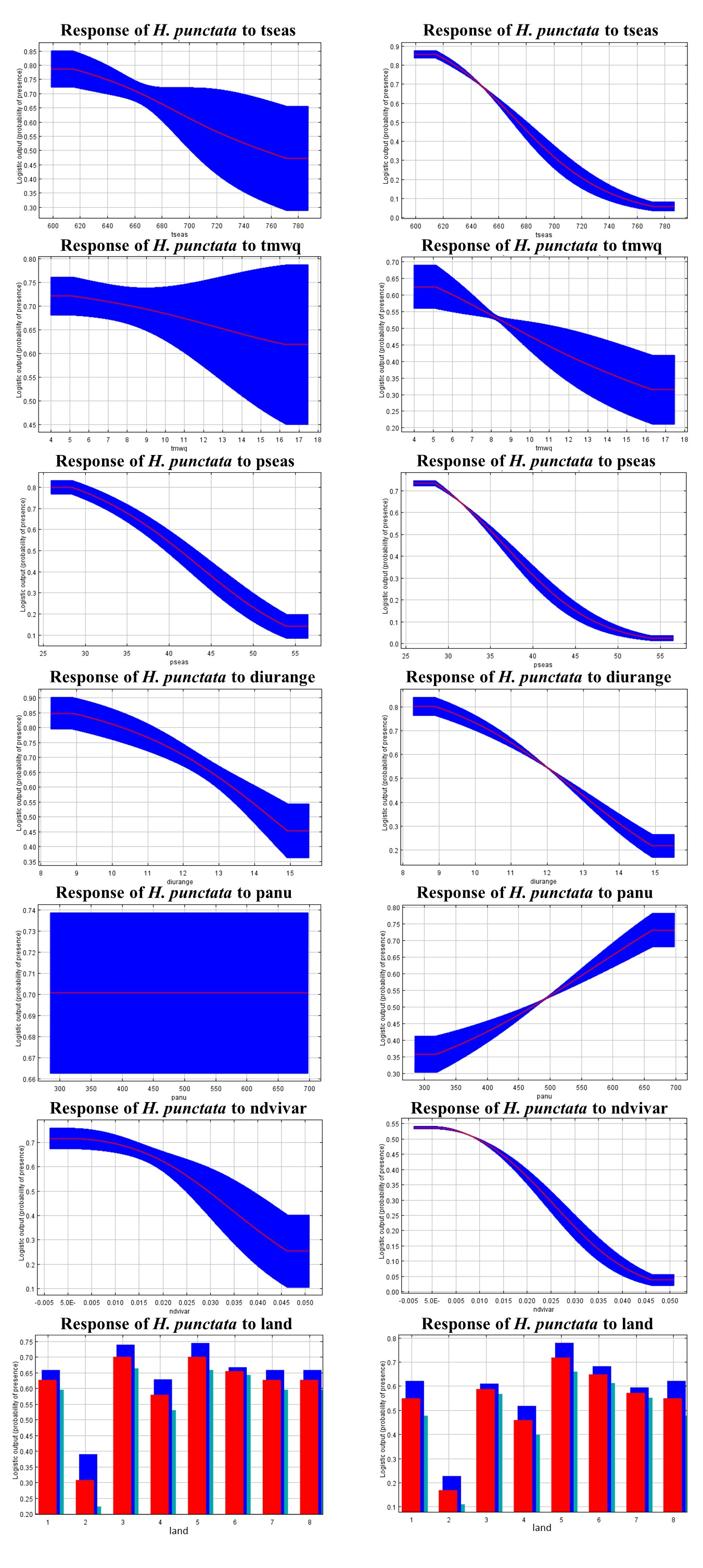

Supplement: S9 Fig — Species response curves characterizing how each predictor influencing the MaxEnt prediction in Haemaphysalis punctata suitability model. Left charts show how the logistic prediction changed as each predictor varied while keeping all other predictors at their average sample value. Right charts show MaxEnt models created using only the corresponding variable. Red lines/bars: mean of the 10 repetitions. Blue area/blue and turquois bars: standard deviation. In the land (categorical predictor) chart (bottom), 1: other land uses; 2: crop; 3: grassland; 4: shrub; 5: deciduous broadleaf forest; 6: evergreen broadleaf forest 7: coniferous forest and 8: woodland. (TIF) [file pntd.0013741.s014.tif]
